# A distinct D1-MSN subpopulation down-regulates dopamine to promote negative emotional state

**DOI:** 10.1038/s41422-021-00588-5

**Published:** 2021-11-30

**Authors:** Zhiyuan Liu, Qiumin Le, Yanbo Lv, Xi Chen, Jian Cui, Yiming Zhou, Deqin Cheng, Chaonan Ma, Xiujuan Su, Lei Xiao, Ruyi Yang, Jiayi Zhang, Lan Ma, Xing Liu

**Affiliations:** Department of Neurosurgery, Huashan Hospital, School of Basic Medical Sciences, and Institutes of Brain Science, State Key Laboratory of Medical Neurobiology and MOE Frontiers Center for Brain Science, Fudan University, Shanghai, China

**Keywords:** Single-molecule biophysics, Mechanisms of disease

## Abstract

Dopamine (DA) level in the nucleus accumbens (NAc) is critical for reward and aversion encoding. DA released from the ventral mesencephalon (VM) DAergic neurons increases the excitability of VM-projecting D1-dopamine receptor-expressing medium spiny neurons (D1-MSNs) in the NAc to enhance DA release and augment rewards. However, how such a DA positive feedback loop is regulated to maintain DA homeostasis and reward-aversion balance remains elusive. Here we report that the ventral pallidum (VP) projection of NAc D1-MSNs (D1^NAc-VP^) is inhibited by rewarding stimuli and activated by aversive stimuli. In contrast to the VM projection of D1-MSN (D1^NAc-VM^), activation of D1^NAc-VP^ projection induces aversion, but not reward. D1^NAc-VP^ MSNs are distinct from the D1^NAc-VM^ MSNs, which exhibit conventional functions of D1-MSNs. Activation of D1^NAc-VP^ projection stimulates VM GABAergic transmission, inhibits VM DAergic neurons, and reduces DA release into the NAc. Thus, D1^NAc-VP^ and D1^NAc-VM^ MSNs cooperatively control NAc dopamine balance and reward-aversion states.

Emotional valence, the positive (rewarding) or negative (aversive) internal state of an animal,^1^ is fundamental for motivated behavior and reinforcement learning.^2^ Dopamine (DA) is a critical mediator for emotional and motivated behaviors. The nucleus accumbens (NAc), which reciprocally innervates the ventral mesencephalon (VM), including the ventral tegmental area (VTA) and substantia nigra (SNc), has been identified as a critical hub for processing and coding information related to reward and aversion.^3–5^ Up to 95% of neurons in the NAc are dopaminoceptive GABAergic medium spiny neurons (MSNs).^6,7^ D1-dopamine receptor-expressing MSNs (D1-MSNs) project directly from the NAc to the VM (the direct pathway), or convey information to the VM indirectly through the innervation of the ventral pallidum (VP) (the indirect pathway). D2-dopamine receptor-expressing MSNs (D2-MSNs) primarily target VP neurons that innervate the VM (the indirect pathway).^8–10^

The prevailing model posits that the activation of D1-MSNs is rewarding and activation of D2-MSNs is aversive.^11,12^ Optogenetic activation of NAc D1-MSNs enhances cocaine-induced conditioned place preference (CPP), whereas optogenetic activation of D2-MSNs suppresses it.^13^ Chemogenetic inhibition of NAc D2-MSNs augments cocaine seeking.^14^ It has been demonstrated that DA release in the NAc activates both D1- and D2-dopamine receptors, and increases cAMP/PKA activity in D1-MSNs and inhibits cAMP/PKA activity in D2-MSNs.^15,16^ Then DA binding to D1- and D2-receptors enhances excitability of D1-MSNs and decreases the excitability of D2-MSNs through the regulation of cAMP/PKA signaling.^15,17^ It was well known that activation of D1 receptor and inactivation of D2 receptors in the NAc produces opposing behavioral effects.^18^ DA release induced activation of D1-MSNs and inhibition of D2-MSNs synergistically augment DA release in the NAc and promote greater reward.^19^ How is this positive feedback loop of DA release and reward regulated? Is there a negative feedback or brake mechanism?

In the current study, we show that activation of VM- and VP-projections of NAc D1-MSNs (D1^NAc-VM^ and D1^NAc-VP^ pathways) is elicited by stimuli of different valences and produces opposing behavioral responses. Contradicting to the conventional role of D1-MSNs, the D1^NAc-VP^ projection is suppressed by a rewarding stimulus and activated by an aversive stimulus, and activation of D1^NAc-VP^ pathway mediates aversive, but not reward response. We also reveal that VP and VM projecting D1-MSNs are two largely separate populations of neurons. Our data indicate that D1^NAc-VP^ and D1^NAc-VM^ projection neurons encode opposing emotional valences via differential regulation of VM DAergic neurons and NAc DA release, cooperatively modulating reward-aversion state.

## Results

### Negative stimulus induces activation of D1^NAc-VP^ projection and concurrent suppression of D1^NAc-VM^ projection, while positive stimulus suppresses D1^NAc-VP^ projection and concurrently activates D1^NAc-VM^ projection

**Fig. 1:**
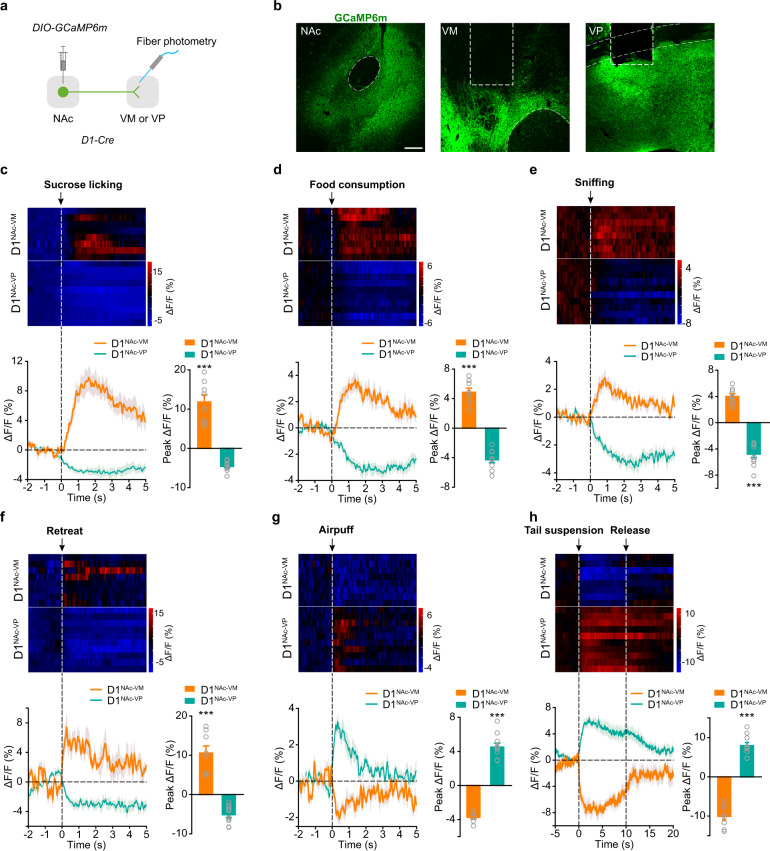
Rewarding and aversive stimuli induce opposing responses in D1^NAc-VM^ and D1^NAc-VP^ projections. **a** Strategy for recording calcium activity of D1^NAc-VM^ or D1^NAc-VP^ projections in response to stimuli in freely moving mice. *AAV*_*9*_*-EF1α-DIO-GCaMP6m* was injected into the NAc of *D1-Cre* mice, with optical fiber implanted over the VM or VP. **b** Representative images showing GCaMP^+^ terminals in the VM and VP. Scale bar: 100 μm. **c**–**h** Heat map (Top), plot and statistical graph (Bottom) of group average GCaMP responses aligned to the onset of sucrose licking (**c**), sucrose pellet consumption (**d**), sniffing of female stranger (**e**), retreat from the novel object in approach-avoidance task (**f**), air puff (**g**), or tail suspension (**h**). In sucrose licking and consumption task, Ca^2+^ signal was recorded when mice were licking and chewing pellet. In approach-avoidance task, when mice encountered a novel object, they approached and explored, and then quickly retreated from it. Ca^2+^ signal was recorded during an approach and a quick retreat followed. In social interaction task, Ca^2+^ signal was recorded during chasing and sniffing the stranger female. In air puff and tail suspension tasks, Ca^2+^ signal was recorded when the air puff was unexpectedly delivered to the eye and the tail was unexpectedly lifted. Bar graph: Quantification of peak amplitude of Ca^2+^ events to and after the onset of sucrose licks, sucrose pellet consumption, sniffing, retreat, air puff, or tail suspension [Mann-Whitney U test: **c** D1^NAc-VM^ *n* = 8, D1^NAc-VP^ *n* = 10, Z = −3.554, *P* = 0.000046; **f** D1^NAc-VM^ *n* = 8, D1^NAc-VP^ *n* = 10, Z = −3.554, *P* = 0.000046; Two-tailed Student’s *t*-test: **d** D1^NAc-VM^ *n* = 8, D1^NAc-VP^ *n* = 10, *t*(16) = 13.552, *P* < 0.001; **e** D1^NAc-VM^ *n* = 8, D1^NAc-VP^ *n* = 10, *t*(16) = 12.049, *P* < 0.001; **g** D1^NAc-VM^ *n* = 8, D1^NAc-VP^ *n* = 10, *t*(16) = −14.66, *P* < 0.001; **h** D1^NAc-VM^ *n* = 8, D1^NAc-VP^ *n* = 10, *t*(16) = −13.856, *P* < 0.001.] ^***^P < 0.001.

To explore the potential roles of projections of NAc D1-MSNs to VM and VP, we first examined their responses to stimuli of positive and negative valences. We expressed GCaMP in the accumbal D1-MSNs and recorded calcium transient in their projections in the VM and VP (Fig. 1a, b). As shown in Fig. 1c, the photometry recording showed that the onset of sucrose licking as detected by lickometer in water-restricted mice triggered an increase of GCaMP fluorescence in D1^NAc-VM^ projection in the VM, but a decrease of GCaMP signal in D1^NAc-VP^ projection in the VP (Fig. 1c). To minimize the possible effect of stress factors resulted from water or food retraction, the response of these two projections to palatable food consumption was examined. Consistent with the result of sucrose liking, increased GCaMP fluorescence in D1^NAc-VM^ projection and decreased GCaMP signal in D1^NAc-VP^ projection were detected during sucrose pellet consumption (Fig. 1d). Social interaction with a female stranger is a natural reward to male adults. We found that during social behavioral tests, sniffing the female stranger increased calcium activity in D1^NAc-VM^ projection, but decreased calcium signal in D1^NAc-VP^ projection (Fig. 1e). These data indicate that natural reward stimuli induce opposing responses in D1^NAc-VM^ and D1^NAc-VP^ MSNs, causing activation of D1^NAc-VM^ and suppression of D1^NAc-VP^ MSNs. Safety is regarded as positive valence and encoded by dopamine neurons.^20^ In approach-avoidance task, when mice encounter a novel object in a familiar environment, they typically approach and explore it due to fear and curiosity, and then quickly retreat from it. Retreat produces safety and reinforcement signal.^21–23^ We observed that when mice began to retreat from the novel object in the center of the open field, GCaMP fluorescence increased rapidly in D1^NAc-VM^ projection, but dropped in D1^NAc-VP^ projection (Fig. 1f), confirming that stimuli of positive valence activate D1^NAc-VM^ MSNs while inhibit D1^NAc-VP^ MSNs. We speculate that D1^NAc-VM^ and D1^NAc-VP^ MSNs may be involved in encoding positive and negative valences, respectively.

Next acute stressors including air puff and tail suspension^24,25^ were introduced as the stimuli of negative valence. In contrast to the response to a natural reward, air puff caused a decrease in GCaMP fluorescence intensity in D1^NAc-VM^ projection and an increase in D1^NAc-VP^ projection (Fig. 1g). Similarly, during tail suspension test, we observed a decrease in GCaMP signal in D1^NAc-VM^ and an increase in D1^NAc-VP^ projections when mice were chased and lifted by hand (Fig. 1h). After mice were released, GCaMP fluorescence returned to the baseline. Control mice expressing eGFP in D1-MSNs showed no significant change in GCaMP fluorescent intensity during these stimulus-induced conditions, suggesting that the observed changes in calcium activity were not movement artifacts (Supplementary information, Fig. S1). Our results show that positive stimuli increase the activity of D1^NAc-VM^ projection and decrease the activity of D1^NAc-VP^ projection; while negative stimuli decrease the activity of D1^NAc-VM^ projection and increase the activity of D1^NAc-VP^ projection. These data demonstrate that D1^NAc-VM^ and D1^NAc-VP^ projections are activated by stimuli of opposite valences and the stimulus induced activation of one projection is concurrent with suppression of another projection. Thus, D1^NAc-VM^ and D1^NAc-VP^ projections may bi-directionally and cooperatively regulate responses to rewarding and aversive stimuli.

### Activation of D1^NAc-VM^ and D1^NAc-VP^ projections lead to opposite emotional states

**Fig. 2:**
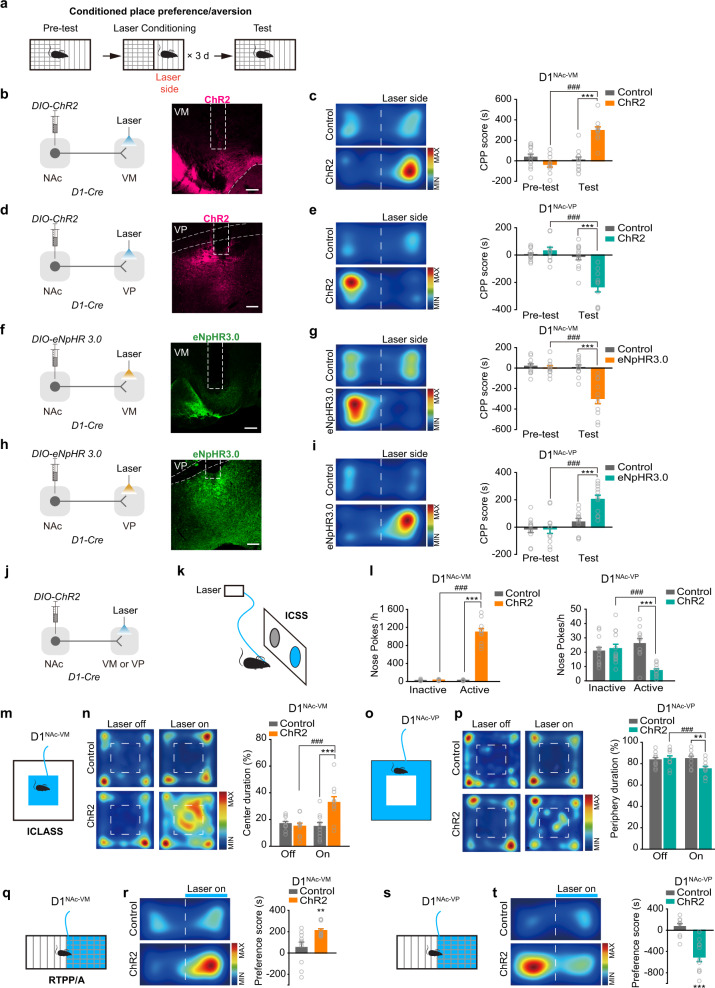
Activation of D1^NAc-VP^ or D1^NAc-VM^ projections drives opposing emotional responses. **a** Schematic of CPP/A task. *AAV*_*9*_*-EF1α-DIO-hChR2-mCherry* or *AAV*_*9*_*-EF1α-DIO-eNpHR3.0-EYFP* was injected into the NAc and an optical fiber was implanted over the VM or VP of *D1-Cre* mice. After three-day laser-paired place conditioning in ChR2 or eNpHR3.0 expressing mice, CPP/A test was performed. **b**, **d**, **f**, **h** Viral infection and representative images of the optical fiber tip in the VM or VP. **c**, **e**, **g**, **i** Representative images of locomotor heat maps of Test and bar graphs of CPP score. [Two-way RM ANOVA: **c** Control *n* = 12, ChR2 *n* = 10, *F*_treatment×session_(1, 43) = 29.361, *P* < 0.001; **e** Control *n* = 12, ChR2 *n* = 10, *F*_treatment×session_(1_,_ 43) = 22.372, *P* < 0.001; **g** Control *n* = 11, eNpHR3.0 *n* = 11, *F*_treatment×session_(1, 43) = 18.787, *P* < 0.001; **i** Control *n* = 11, eNpHR3.0 *n* = 11, *F*_treatme*n*t×session_(1, 43) = 7.475, *P* = 0.013] ***P < 0.001 vs Control, ^###^*P* < 0.001 vs Pre-test. **j** Viral infection. *AAV*_*9*_*-EF1α-DIO-hChR2-mCherry* was injected into the NAc and an optical fiber was implanted over the VM or VP of *D1-Cre* mice. **k** Schematic of ICSS task. Mice received optogenetic self-stimulation of D1^NAc-VM^ or D1^NAc-VP^ projection in response to nose-poke. **l** Bar graph of nose-pokes for optical stimulation of D1^NAc-VM^ or D1^NAc-VP^ projection. [RM ANOVA with Geisser-Greenhouse correction, D1^NAc-VM^: Control *n* = 14, ChR2 *n* = 10, *F*_virus×nosepoke_(1, 22) = 215.079, *P* < 0.001; Two-way RM ANOVA_,_ D1^NAc-VP^: Control *n* = 13, ChR2 *n* = 11, *F*_virus×nosepoke_(1, 47) = 29.806, *P* = 0.000017] ***P < 0.001 vs Control, ^###^*P* < 0.001 vs Inactive portal. **m**, **o** Schematic of ICLASS task. When mice entered the marked area (blue), laser was passed to the VM or VP. **n**, **p** Representative locomotor heat maps of ICLASS and bar graph of percentage of duration in the area with laser stimulation. [Two-way RM ANOVA: **n** Control *n* = 10, ChR2 *n* = 10, *F*_treatment×session_ (1, 39) = 10.465, *P* = 0.005; **p** Control *n* = 10, ChR2 *n* = 10, *F*_treatment×session_(1, 39) = 9.502, *P* = 0.006] **P < 0.01, ***P < 0.001 vs Control, ^###^*P* < 0.001 vs Off. **q**, **s** Schematic of RTPP/A task. When mice entered the marked chamber (blue), laser was passed to the VM or VP. **r**, **t** Representative locomotor heat maps of RTPP/A and bar graphs of Preference score. [Two-tailed Student’s *t*-test: **r** Control *n* = 10, ChR2 *n* = 12 *t* (20) = −3.127, *P* = 0.005; Mann-Whitney U test, **t** Control *n* = 10, ChR2 *n* = 11, Z = −3.592, *P* = 0.000068.] **P < 0.01 and ***P < 0.001.

Based on the above results, we raise the question of whether activation of D1^NAc-VP^ projection induces the same emotional states as activation of D1^NAc-VM^ projection does. We conditioned mice by laser stimulation of D1^NAc-VM^ or D1^NAc-VP^ projection in a place preference apparatus (Fig. 2a). As expected, after laser conditioning, D1^NAc-VM^:ChR2 mice developed a preference for the laser side (Fig. 2b, c; Supplementary information, Fig. S2a); however, D1^NAc-VP^:ChR2 mice, on the contrary, exhibited a significant aversion to the laser side (Fig. 2d, e). Consistently, D1^NAc-VM^:eNpHR3.0 mice showed a place aversion (Fig. 2f, g; Supplementary information, Fig. S2b), and D1^NAc-VP^:eNpHR3.0 mice developed a preference for the laser conditioned side (Fig. 2h, i). These data indicate that activation of D1^NAc-VP^ projection promotes aversive emotion, opposite to the rewarding effect by activation of D1^NAc-VM^ projection.

Emotional valence is crucial for reinforcement learning. We expressed ChR2 in D1-MSNs, then performed optogenetically intracranial self-stimulation assay (ICSS), intracranial light administration in specific subarea assay (ICLASS), and real-time place preference/avoidance (RTPP/A) task, in which an active nose poke or entering the defined area would trigger laser stimulation of D1^NAc-VM^ or D1^NAc-VP^ projection (Fig. 2j–t). In ICSS task, we observed a robust increase of active nose poke coupled to optogenetic activation of D1^NAc-VM^ projection, and a significantly reduced nose poke coupled to optogenetic activation of D1^NAc-VP^ projection (Fig. 2j–l). In ICLASS task, optogenetic activation of D1^NAc-VM^ projection when mice stayed in the center extended the duration in the center area, while optogenetic activation of D1^NAc-VP^ projection when they stayed in the periphery reduced time spent in this area (Fig. 2m–p). Consistently, in the RTPP/A task, optogenetic activation of D1^NAc-VM^ projection induced the preference for the laser stimulation side, while optogenetic activation of D1^NAc-VP^ projection induced avoidance for the laser side (Fig. 2q–t). These results indicate that activation of D1^NAc-VM^ projection drives positive reinforcement and activation of D1^NAc-VP^ projection promotes negative reinforcement.

**Fig. 3:**
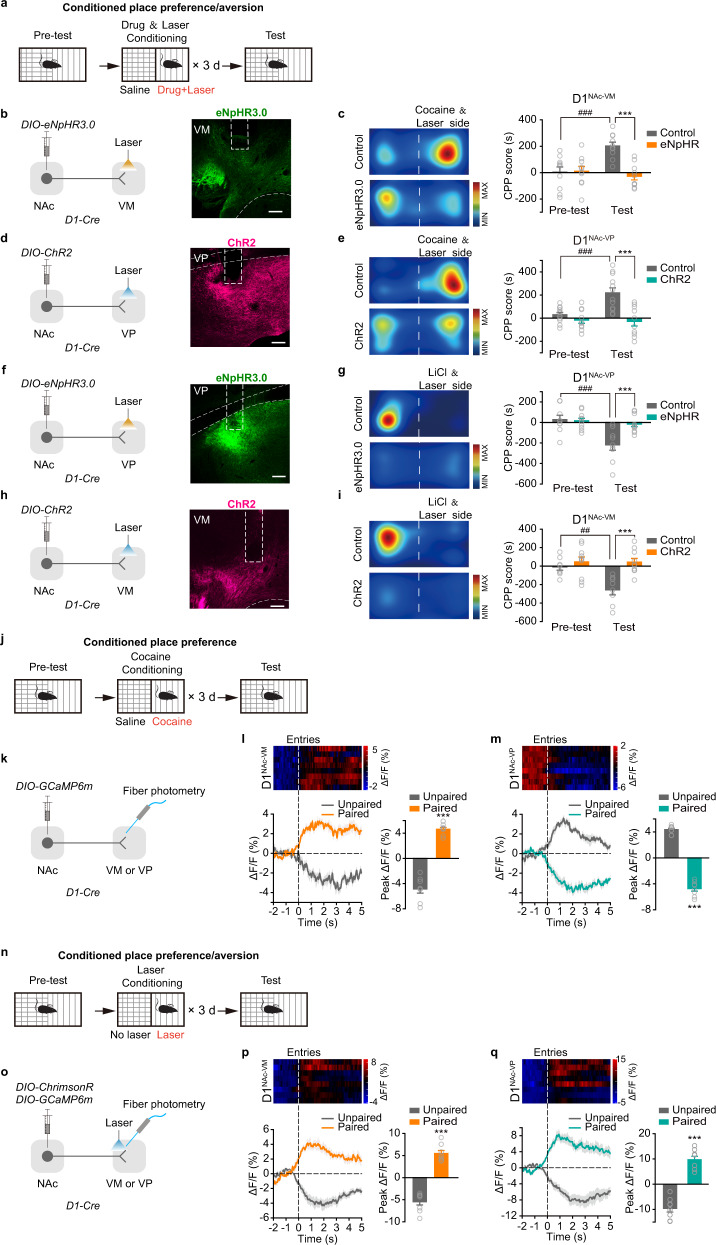
Activation of D1^NAc-VP^ projection inhibits cocaine CPP and activation of D1^NAc-VM^ projection inhibits LiCl CPA. **a** Schematic of experimental design. Laser stimulation was presented during cocaine- or LiCl-place conditioning. After three-day conditioning, the CPP/A test was performed. **b**, **d**, **f**, **h** Viral infection and representative images. *AAV*_*9*_*-EF1α-DIO-hChR2-mCherry*, *AAV*_*9*_*-EF1α-DIO-mCherry*, *AAV*_*9*_*-EF1α-DIO-eNpHR3.0-EYFP*, or *AAV*_*9*_*-EF1α-DIO-EYFP* was injected into the NAc of *D1-Cre* mice, and optical fibers were bilaterally implanted over the VM or VP. Scale bar: 100 μm. **c**, **e**, **g**, **i** Representative images of locomotor heat maps of Test and bar graphs of CPP score. [Two-way RM ANOVA: **c** Control *n* = 10, eNpHR3.0 *n* = 10, *F*_treatment×session_(1, 39) = 13.823, *P* = 0.002; **e** Control *n* = 12, ChR2 *n* = 10, *F*_treatment×session_(1, 43) = 8.751, *P* = 0.008; **g** Control *n* = 9, eNpHR *n* = 11, *F*_treatment×session_(1, 39) = 7.403, *P* = 0.014; **i** Control *n* = 8, ChR2 *n* = 10, *F*_treatment×session_(1, 35) = 6.243, *P* = 0.024] **P < 0.01,***P < 0.001 vs Control, ^###^*P* < 0.001 vs Pre-test. **j** Schematic of experimental design. After three-day cocaine-conditioning, photometry recording of Ca^2+^ transient of D1^NAc-VM^ and D1^NAc-VP^ projections during CPP test was performed. **k** Viral infection. *AAV*_*9*_*-EF1α-DIO-GCaMP6m* was injected into the NAc of *D1-Cre* mice, with optical fiber implanted over the VM or VP. **l**, **m** (Top) Representative heat map of Ca^2+^ signaling in D1^NAc-VM^ and D1^NAc-VP^ projection during successive entries into cocaine paired side during CPP test. (Bottom) Averaged Ca^2+^ traces and quantification of peak amplitude of Ca^2+^ events around entry [Two-tailed Student’s *t*-test, D1^NAc-VM^ *n* = 8, *t*(14) = −12.858, *P* < 0.001; D1^NAc-VP^ *n* = 8, *t*(14) = 20.054, *P* < 0.001] ***P < 0.001. **n** Schematic of experimental design. After three-day laser-conditioning, photometry recording of Ca^2+^ transient of D1^NAc-VM^ and D1^NAc-VP^ projections during CPP/A test was performed. **o** Viral infection. *AAV*_*9*_*-EF1α-DIO-GCaMP6m* and *AAV*_*9*_*-hSyn-FLEX-ChrimsonR-tdTomato* were injected into the NAc of *D1-Cre* mice, with optical fiber implanted over the VM or VP. **p**, **q** (Top) Representative heat map of Ca^2+^ signaling in D1^NAc-VM^ and D1^NAc-VP^ projections during successive entries into optical stimulation paired side and the opposite side during CPP/A test. (Bottom) Averaged Ca^2+^ traces and quantification of peak amplitude of Ca^2+^ events around entry [Two-tailed Student’s *t*-test, D1^NAc-VM^ *n* = 8, *t*(14) = −10.788, *P* < 0.001; D1^NAc-VP^ *n* = 8, *t*(14) = −9.615, *P* < 0.001] ***P < 0.001.

To further confirm the roles of these two projections in the regulation of emotional states, we applied optogenetic stimulation of these projections in cocaine-induced reward and LiCl-induced aversion models (Fig. 3a). The data showed that optogenetic inhibition of D1^NAc-VM^ projection and optogenetic activation of D1^NAc-VP^ projection during cocaine conditioning could both reduce cocaine CPP (Fig. 3b–e), and optogenetic inhibition of D1^NAc-VP^ projection and optogenetic activation of D1^NAc-VM^ projection during LiCl conditioning could both suppress LiCl CPA (Fig. 3f–i). Moreover, when the mice were conditioned with stimulation of D1^NAc-VM^ projection on one side and injection of cocaine on the other side of the CPP chamber, they showed no preference for either side (Supplementary information, Fig. S3a, b), confirming that activation of D1^NAc-VM^ projection produces comparable rewarding effects as cocaine injection. In CPA test, mice conditioned with stimulation of D1^NAc-VP^ projection on one side and LiCl injection on the other side exhibited ~40 % reduced aversion to the optical stimulation side compared with stimulation of D1^NAc-VP^ projection alone (Supplementary information, Fig. S3c, d), suggesting the aversion produced by direct activation of D1^NAc-VP^ projection is stronger than that evoked by systematic injection of LiCl. These data further demonstrate that activation of D1^NAc-VP^ projection or inhibition of D1^NAc-VM^ projection induces aversion, while activation of D1^NAc-VM^ projection or inhibition of D1^NAc-VP^ projection promotes reward, indicating that these two projections drive opposing valence states and may cooperatively regulate rewarding and aversion.

Next, we did photometry recording of Ca^2+^ transient of D1^NAc-VM^ and D1^NAc-VP^ projections during the test phase of cocaine CPP and optical stimulation-induced CPP/A (Fig. 3j–q). The overall frequency of Ca^2+^ transient events in VM and VP axonal terminals of D1-MSNs was both significantly elevated during cocaine conditioning (Supplementary information, Fig. S3e–i). In cocaine CPP test, the GCaMP signal was increased in D1^NAc-VM^ projection and decreased in D1^NAc-VP^ projection when mice entered the cocaine-paired side (Fig. 3j–m). Moreover, during test of CPP/A induced by stimulation of ChR2 expressing D1^NAc-VM^ or D1^NAc-VP^ projection, the GCaMP signal in D1^NAc-VM^ projection was increased when mice that had acquired CPP entered the stimulation-paired side (Fig. 3n–p), and the GCaMP signal in D1^NAc-VP^ projection was increased when mice that had acquired CPA entered the stimulation-paired side (Fig. 3q).

### VP- and VM-projecting NAc D1-MSNs exhibit distinct anatomic, molecular, and electrophysiological properties

**Fig. 4:**
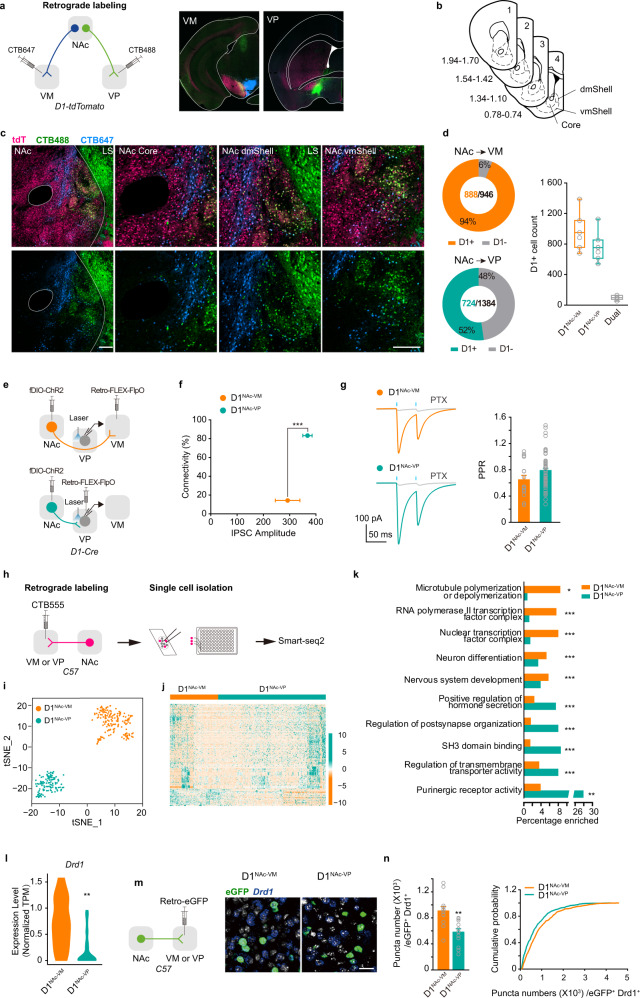
D1^NAc-VM^ and D1^NAc-VP^ neurons are two populations with distinct anatomic, molecular, and electrophysiological features. **a** Schematic of CTB labeling and confocal images for injections of CTB647 into the VM and CTB488 into the VP of *D1-tdTomato* mice. **b** Schemtic of sites imaged for quantification across the rostro-caudal gradient of NAc. **c** Confocal images for CTB labeled neurons in the NAc core, dmShell, and vmShell. Scale bar: 100 μm. **d** Left: quantification of CTB and tdTomato (D1-MSNs) overlap in the NAc. Right: summary of the number of CTB647^+^ D1-MSNs, CTB488^+^ D1-MSNs and double positive D1-MSN in the NAc [CTB647: 888 ± 89 cells, CTB488:724 ± 81 cells of 4 slices per mouse]. **e** Schematic of experimental design. Retrograde *AAV*_*2/retro*_*-DIO-FlpO* was injected in the VP or VM, combined with the injection of *AAV9-EF1α-fDIO-ChR2-mCherry* into the NAc of *D1-Cre* mice. **f** Connectivity plot summarizing optogenetic circuit mapping. In the VP, 83.3% of neurons were innervated by D1^NAc-VP^ neurons (66 cells from 6 mice; average connectivity strength: 367.77 ± 17.66 pA), whereas only 14.3% VP neurons received innervation from D1^NAc-VM^ neurons (112 cells from 8 mice; average connectivity strength: 292.13 ± 46.74 pA). Connectivity rate, Pearson χ2 ratio, 26.670, *P* < 0.001. **g** Representative responses to optogenetic stimulation (blue bars) of D1^NAc-VM^ and D1^NAc-VP^ projections in the VP. Currents were blocked by picrotoxin (PTX: 20 mM) [D1^NAc-VM^, 0.65 ± 0.07; D1^NAc-VP^, 0.79 ± 0.04; *t*(69) = −1.852, *P* = 0.0683]. **h** Workflow for single cell sequencing of D1^NAc-VM^ and D1^NAc-VP^ neurons retrogradelly labeled by CTB. **i** Unbiased clustering of D1-expressing neurons using t-SNE according to their whole-transcriptome correlation distance. Each cell is represented as a dot and colored by a clustering algorithm. **j** Heatmap of the differentially expressed genes between the two populations. Each column represents a single cell, and each row represents a single gene. The genes are ordered by hierarchical clustering the expression difference. Color represents the expression *Z*-score of the cells. **k** Gene ontology overrepresentation analysis of D1^NAc-VM^ and D1^NAc-VP^ populations. Bar length represents the number of genes differentially expressed in the population/the number of genes that are expressed and annotated to the pathway [Fisher’s exact test, see Supplementary information, Table S1]. *P < 0.05, **P < 0.01, ***P < 0.001. **l** Bar graph showing the expression of *Drd1* in D1^NAc-VM^ and D1^NAc-VP^ neurons [Negative binomial generalized linear models, D1^NAc-VM^ *n* = 120 cells, D1^NAc-VP^ *n* = 182 cells, W = 2.607, *P* = 0.009.] **P < 0.01. **m** Viral infection and representative confocal images of eGFP and *Drd1* expression in the NAc. C57 mice were injected with *AAV*_*2/retro*_*-hSyn-eGFP* into the VP or VM. Then smFISH was performed with *Drd1* in the brain slice containing the NAc. Scale bar: 25 μm. **n** Quantification bar graph and cumulative frequency distribution of *Drd1* expression in D1^NAc-VM^ and D1^NAc-VP^ neurons (*Drd1*^+^ eGFP^+^). [Two-tailed Student’s *t*-test, *t*(23) = 3.757, *P* = 0.001; Kolmogorov-Smirnov test, *P* < 0.001.] **P < 0.01, ***P < 0.001.

To definitively examine the anatomical organization of D1^NAc-VM^ and D1^NAc-VP^ projectors, we used cholera-toxin subunit B (CTB) to label D1^NAc-VM^ and D1^NAc-VP^ neurons in individual mouse as previously reported.^26^ We injected CTB488 into the VP and CTB647 into VM of *D1-tdTomato* mice (Fig. 4a). D1^NAc-VM^ and D1^NAc-VP^ neuronal populations were distributed in the whole NAc, including the core and shell (Fig. 4b, c). Overall, equivalent numbers of tdTomato^+^ (D1-MSN) cells in the NAc were labeled by CTB647 and CTB488. 94% of CTB647-labeled cells were D1^NAc-VM^ projectors and 52% of CTB488-labeled cells were D1^NAc-VP^ projectors (Fig. 4d). 6.7% ± 0.4% of labeled cells contained both CTB488 and CTB647 fluorophores (Supplementary information, Fig. S4a–f). These results were confirmed by retrograde tracing with CTB injected into the VP and VM of *D2-eGFP* mice (Supplementary information, Fig. S4g–j). 5.9% ± 0.8% of labeled cells contained both CTB fluorophores (Supplementary information, Fig. S4i). We also verified the above results with the retrograde AAV strategy. We injected retrograde AAV encoding flp recombinase in the VP and retrograde AAV encoding Cre recombinase in the VM, and a mixture of AAV encoding Cre-dependent tdTomato and flp-dependent eGFP in the NAc.^27^ Then we carried out multiplexed single-molecule RNA fluorescence in situ hybridization (smFISH) of *drd1* (Supplementary information, Fig. S4k, l). Among *drd1*^+^ neurons, 91.7% of eGFP^+^ cells were D1^NAc-VM^ projectors and 55.9% of tdTomato^+^ cells were D1^NAc-VP^ projectors. 5.8% ± 1.1% of labeled cells expressed both tdTomato and eGFP in the NAc (Supplementary information, Fig. S4m–o). Moreover, rabies virus-based monosynaptic tracing^28–30^ revealed that D1^NAc-VP^ neurons received more inputs from the BLA and thalamus, and D1^NAc-VM^ neurons received more inputs from the PrL and IL (Supplementary information, Fig. S5a–c).

We next assayed the functional connectivity of D1^NAc-VM^ and D1^NAc-VP^ projectors to the VP by injecting retrograde AAV encoding Cre-dependent flp recombinase in the VP or VM, and AAV encoding flp dependent ChR2 in the NAc of *D1-Cre* mice. We used optical stimulation of D1^NAc-VM^ and D1^NAc-VP^ projectors in the VP to evoke GABAergic inhibitory postsynaptic currents (oIPSC) and found that 83.3% of VP cells received innervation from D1^NAc-VP^ projectors, while 14.3% of the VP cells were innervated by D1^NAc-VM^ projectors (Fig. 4e, f). The currents were GABA_A_ mediated, as they could be blocked by picrotoxin (PTX) (Fig. 4g). These results suggest that the connectivity rate of D1^NAc-VP^ synapses onto the VP neurons is greater than that of D1^NAc-VM^ synapses. In addition, chemogenetic or optogenetic activation of D1^NAc-VM^ neurons by CNO and laser-induced a preference for CNO- or laser-paired side, while chemogenetic or optogenetic activation of D1^NAc-VP^ neurons induced an avoidance of CNO- or laser-paired side (Supplementary information, Figs. S2c, d and S6). Together, data from these experiments indicate that D1^NAc-VM^ and D1^NAc-VP^ projectors are largely two populations within differential neural circuits, providing an anatomical basis for the distinct roles in valence encoding.

We next examined the molecular profiles of D1^NAc-VM^ and D1^NAc-VP^ populations by retrograde labeling of VM- or VP-projecting NAc neurons with CTB555 and single-cell transcriptome sequencing (Fig. 4h; Supplementary information, Fig. S7a–g). Transcriptome analysis of *Drd1* expressing CTB555^+^ cells showed that D1^NAc-VM^ and D1^NAc-VP^ populations were nicely separated in t-SNE plots (Fig. 4i). We compared differentially expressed genes between the two populations. By assigning a *P* value ≤ 0.05 for the level of expression as the cutoff, we detected 779 genes exhibiting enrichment in D1^NAc-VP^ population and 132 genes more abundant in D1^NAc-VM^ population (Fig. 4j). Among these genes, the differential expression of *Arc* and *Klf5* in D1^NAc-VM^ and D1^NAc-VP^ populations were also verified by smFISH (Supplementary information, Fig. S7h–k). Gene ontology enrichment of D1^NAc-VM^ and D1^NAc-VP^ populations by gene over representation analysis revealed that D1^NAc-VP^ population is distinct from D1^NAc-VM^ population in purinergic receptor signaling and transmembrane transporter activity, while D1^NAc-VM^ populations showed distinct enrichment in pathways such as transcription regulation and nervous system development (Fig. 4k). Notably, the transcript level of *Drd1*, the gene encoding dopamine D1 receptor, was significantly lower in D1^NAc-VP^ neurons than D1^NAc-VM^ neurons (Fig. 4l), and this was confirmed by smFISH of *Drd1* combined with AAV-based retrograde labeling (eGFP^+^) as well (Fig. 4m, n). *Pdyn*, *Tac1*, *Chrm4*, *Foxp1*, *Isl1*, and *Slc35d3* are selectively expressed in D1-MSNs, and *Adora2a*, *Sp9*, *Penk*, *Gpr52*, and *Adk* are selectively expressed in D2-MSNs.^31–33^ We examined the expression of these D1-MSN and D2-MSN markers in D1^NAc-VM^ and D1^NAc-VP^ neurons. We found that the percentage of *Tac1* and *Pdyn* positive D1^NAc-VM^ neurons was greater than that of D1^NAc-VP^ neurons (Supplementary information, Fig. S8a, c), and the expression levels of *Tac1* and *Pdyn* in D1^NAc-VM^ neurons were also higher than those in D1^NAc-VP^ neurons (Supplementary information, Fig. S8b).

The molecular features of D1^NAc-VM^ and D1^NAc-VP^ neurons, and their distinct anatomic properties may posit their differential intrinsic excitability and synaptic plasticity. By whole-cell patch-clamp recording, we found that the intrinsic excitability of D1^NAc-VP^ neurons was greater than that of D1^NAc-VM^ neurons but lower than that of D2^NAc-VP^ neurons (Supplementary information, Fig. S9a–d). The frequency of mIPSC of D1^NAc-VP^ neurons was lower than that of D1^NAc-VM^ neurons but higher than that of D2^NAc-VP^ neurons (Supplementary information, Fig. S9e–f). The frequency and amplitude of mEPSC were not different among them (Supplementary information, Fig. 9e–f). These results suggest that the basal presynaptic GABA release onto D1^NAc-VP^ neurons is weaker than that onto D1^NAc-VM^ neurons, resulting in stronger inhibition of D1^NAc-VM^ neurons relative to D1^NAc-VP^ neurons.

### Activation of D1^NAc-VP^ projection inhibits VM DA neurons via VM GABAergic neurons

**Fig. 5:**
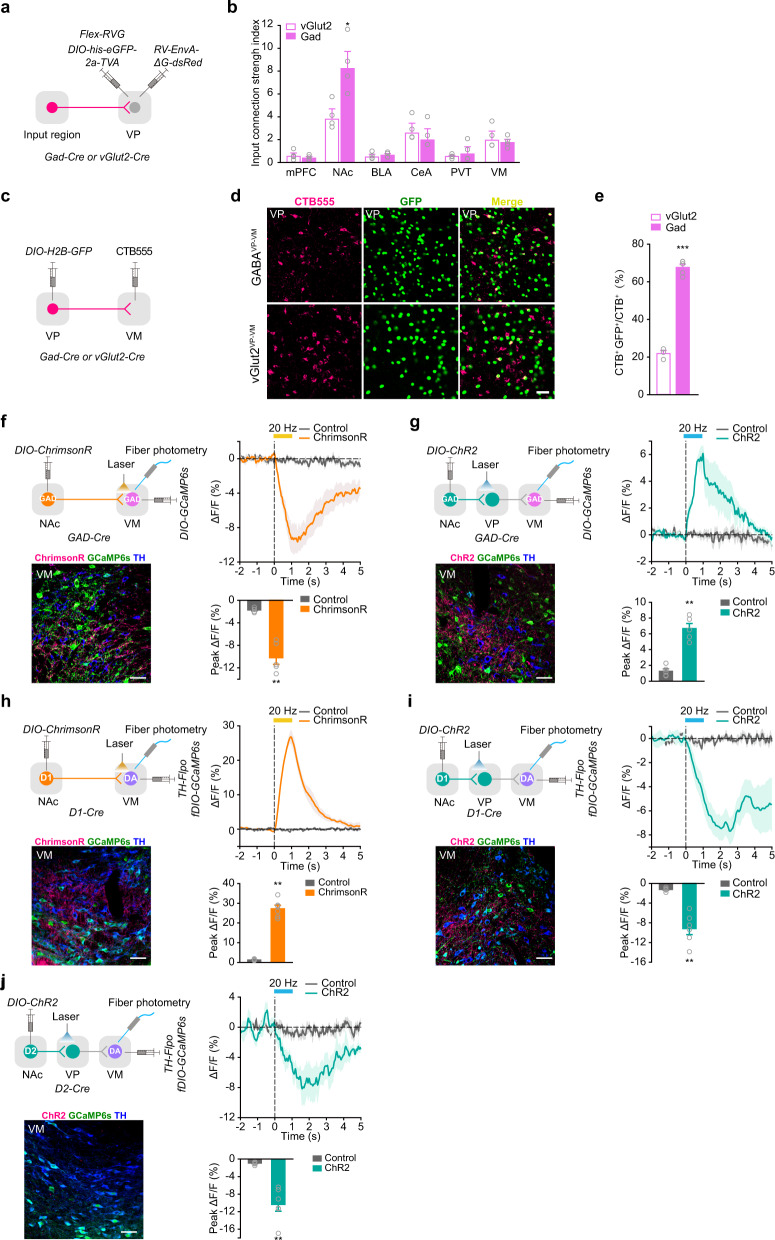
D1^NAc-VM^ and D1^NAc-VP^ pathways drive opposing regulation of VM DAergic and GABAergic neurons. **a**, **b **Schematic of rabies virus-based monosynaptic tracing (**a**) and quantification of input connection strength index (**b**). *RV-ENVA-deltaG-dsRed* (RV*d*G) was injected into the VP 2-week after unilateral injection of *AAV*_*9*_*-EF1α-DIO-his-eGFP-2a-TVA*, and *AAV*_*9*_*-EF1α-DIO-RVG* into the VP of *Gad-Cre* and *vGlut2-Cre* mice. Numbers of labeled presynaptic neurons/numbers of starter neurons were plotted [Two-tailed Student’s *t*-test, Gad *n* = 4, vGlut2 *n* = 4, NAc *t*(6) = 3.318, *P* = 0.016] *P < 0.05. **c**–**e** CTB retrograde labeling of GABA^VP-VM^ and vGlut2^VP-VM^ neurons. CTB555 was injected into the VM and *AAV*_*9*_*-EF1α-DIO-H2B-GFP* was injected into the VP of *Gad-Cre* or *vGlut2-Cre* mice (**c**). Representative images of retrograde labeling (**d**). Percentage of GABA^VP-VM^ and vGlut2^VP-VM^ neurons (**e**) [Two-tailed Student’s *t*-test, Gad *n* = 5, vGlut2 *n* = 3, *t*(6) = −17.902, *P* = 0.000002.] ***P < 0.001. **f**–**j** Optical fibers were implanted in the VM/VP and VM to exert laser stimulation and record CaMP6s signal simultaneously. Confocal images show ChrimsonR^+^ or ChR2^+^ terminals (Red), GCaMP6s^+^ cell bodies (Green), and TH^+^ neurons (Blue) in the VM. Scale bar: 50 μm. Statistical graph of group average GCaMP responses aligned to the onset of optical stimulation and bar graph of peak response to optical stimulation at 20 Hz. *AAV*_*9*_*-hSyn-FLEX-ChrimsonR-tdTomato* or *AAV*_*9*_*-EF1α-DIO-hChR2-mCherry* was injected into the NAc and *AAV*_*9*_*-EF1α-DIO-GCaMP6s* was infected in the VM of *Gad-Cre* mice. Calcium activity of VM GABAergic neurons in response to optical stimulation of GABA^NAc-VM^ or GABA^NAc-VP^ projection was recorded (**f**, **g**). [**f** Control *n* = 5, ChrimsonR *n* = 5, Two-tailed Student’s *t*-test, *t*(8) = 7.014, *P* = 0.000111; **g** Control *n* = 5, ChR2 *n* = 5, Mann-Whitney U test, Z = 2.611, *P* = 0.0079] **P < 0.01 and ***P < 0.001. *AAV*_*9*_*-hSyn-FLEX-ChrimsonR-tdTomato or AAV*_*9*_*-EF1α-DIO-hChR2-mCherry* was infected into the NAc and *AAV*_*9*_*-TH-FlpO* and *AAV*_*9*_*-EF1α-fDIO-GCaMP6s* were injected into the VM of *D1-Cre* mice. Activity of VM DAergic neurons in response to optical stimulation of D1^NAc-VM^ or D1^NAc-VP^ projection was recorded (**h**, **i**). [Mann-Whitney U test: **h** Control *n* = 6, ChrimsonR *n* = 6, Z = 2.882, *P* = 0.0022; **i** Control *n* = 6, ChR2 *n* = 6, Z = −2.882, *P* = 0.0022] **P < 0.01. (**j**) *AAV*_*9*_*-EF1α-DIO-hChR2-mCherry* was injected into the NAc and *AAV*_*9*_*-TH-FlpO* and *AAV*_*9*_*-EF1α-fDIO-GCaMP6s* were injected into the VM of *D2-Cre* mice. Activity of VM DAergic neurons in response to optical stimulation of D2^NAc-VP^ projection was recorded. [Mann-Whitney U test, Control *n* = 6, ChR2 *n* = 6, Z = −2.882, *P* = 0.0022] **P < 0.01.

With rabies retrograde labeling, we found that the monosynaptic inputs from the NAc onto VP GABAergic neurons were greater than those onto VP glutamatergic neurons (Fig. 5a, b; Supplementary information, Fig. S10a, b). By injection of CTB555 into the VM and labeling GABA^VP^ or Glu^VP^ neurons with H2B-GFP, we observed that CTB labeled more VM-projecting GABA^VP^ neurons than VM-projecting Glu^VP^ neurons (Fig. 5c–e). These data indicate that GABAergic neurons in the VP provide a major input to VM neurons.

The activity of DAergic neurons is regulated by local VM GABAergic neurons.^34,35^ To assess the impact of D1^NAc-VP^ and D1^NAc-VM^ projections onto mesolimbic dopaminergic neurons, we examined the net effect of optical stimulation of these projections on the activity of VM GABAergic and DAergic neurons where NAc and VP projections were both located (Supplementary information, Fig. S10c, d). Selective activation of GABA^NAc-VM^ projection (ChrimsonR^+^) induced a decrease, while activation of GABA^NAc-VP^ projection (ChR2^+^) induced an increase in the GCaMP fluorescence in VM GABAergic neurons (Fig. 5f, g). Activation of D1^NAc-VM^ projection (ChrimsonR^+^) induced an increase, while activation of D1^NAc-VP^ projection (ChR2^+^) elicited a decrease in GCaMP fluorescence in VM DAergic neurons (Fig. 5h, i). Similar to the stimulation of D1^NAc-VP^ projection, activation of D2^NAc-VP^ projection (ChR2^+^) resulted in a decrease in GCaMP fluorescence in VM DAergic neurons (Fig. 5j). These data suggest that activation of D1^NAc-VM^ projection inhibits VM GABAergic neurons and in turn activates VM DAergic neurons, while activation of NAc-VP projection, including the projections from both D1- and D2-MSN, increases the activity of VM GABAergic neurons and thus decreases the activity of VM DAergic neurons.

**Fig. 6:**
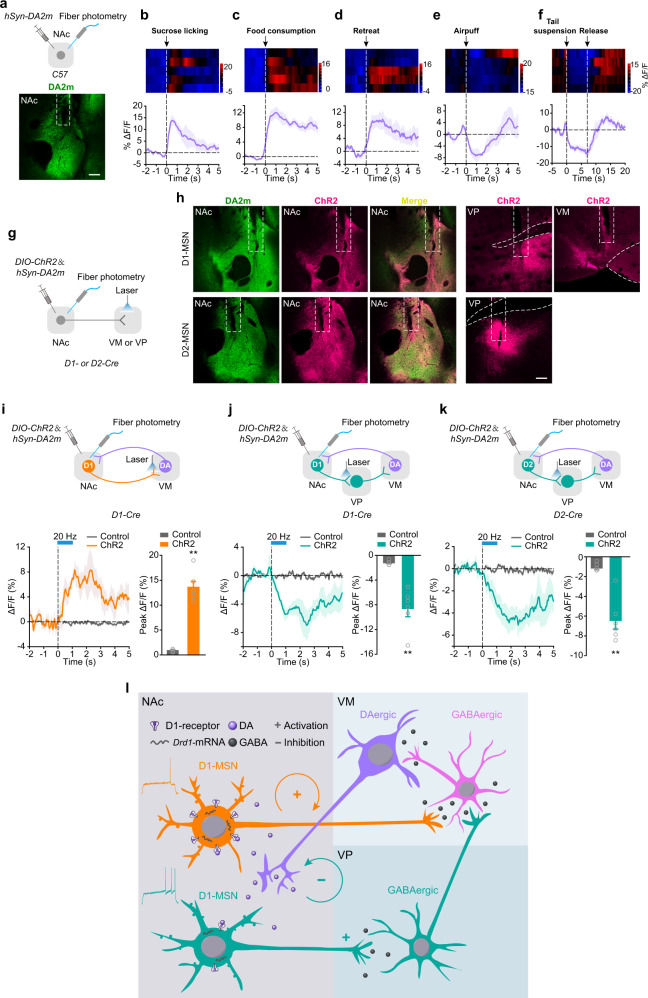
Activation of D1^NAc-VM^ pathway increases DA release into the NAc and activation of D1^NAc-VP^ pathway produces opposing effect. **a**–**f** *AAV-hSyn-DA2m* was infected into the NAc of C57BL/6 mice and an optical fiber was implanted over the NAc to record change of fluorescence of DA sensor DA2m. **a** Viral infection and representative image of DA2m expression and the location of optical fiber tip in the NAc. **b**–**f** Heat map and statistical plot of grouped average DA2m transient for 5% sucrose licking, sucrose pellet consumption, retreat from the novel object in the approaching-retreat test, unpredictable delivery of air puff, and tail suspension (*n* = 5 mice). **g** Optical fibers were implanted in the VM/VP and NAc to exert laser stimulation on D1^NAc-VP^, D1^NAc-VM^, or D2^NAc-VP^ projection and record DA2m signal in the NAc simultaneously. *AAV*_*9*_*-EF1α-DIO-hChR2-mCherry* and *AAV-hSyn-DA2m* were infected into the NAc of *D1-Cre* or *D2-Cre* mice. **h** Representative images of DA2m expression and fiber tip location in NAc, and ChR2 labeled axons in the VP and VM. Left: Statistical graph of group average GCaMP responses aligned to optical stimulation (473 nm, 20 Hz, 1 s) of D1^NAc-VM^ (**i**), D1^NAc-VP^ (**j**), or D2^NAc-VP^ (**k**) projection. Right: bar graph of peak response to optical stimulation [Mann-Whitney U test: **i** Control *n* = 5, ChrimsonR *n* = 6, Z = 2.739, *P* = 0.0043; **j** Control *n* = 5, ChR2 *n* = 6, Z = -2.739, *P* = 0.0043; **k** Control *n* = 6, ChR2 *n* = 6, Z = -2.882, *P* = 0.0022;] **P < 0.01. **l** Working model illustrating mesolimbic D1^NAc-VM^ and D1^NAc-VP^ pathways cooperatively control dopamine balance and reward-aversion state.

The NAc receives DAergic projection from the VM. To directly probe DA release in the NAc, we recorded fluorescence dynamics of DA2m,^36,37^ a newly developed DA sensor, in the NAc upon reward and aversive stimuli (Fig. 6a). When mice voluntarily licked sucrose (5%), ate palatable food, or retreated from the novel object in the approach-avoidance task, we observed a concordant increase of DA sensor fluorescence (Fig. 6b–d). In contrast, air puff and tail suspension both reduced basal DA sensor fluorescence (Fig. 6e, f), reflecting bidirectional changes of local DA release in response to reward and aversion. Next, we monitored DA release in response to the optical manipulation of the activity of D1^NAc-VM^, D1^NAc-VP^, or D2^NAc-VP^ projections (Fig. 6g–k). We detected enhanced DA level in response to optical activation of D1^NAc-VM^ projection (Fig. 6i), and a reduction in response to optical activation of D1^NAc-VP^ and D2^NAc-VP^ projection (Fig. 6j, k). The results above indicate that activation of D1^NAc-VM^ projection promotes DA release and activation of D1^NAc-VP^ projection reduces DA release into the NAc, suggesting that these two subpopulations of D1-MSNs work cooperatively to control the balance of dopamine and reward-aversion states.

## Discussion

In this study, we find that D1^NAc-VM^ and D1^NAc-VP^ neurons are two largely separate populations of D1-MSNs with distinct molecular profiling, specific neural circuits, and membrane excitability. Activation of D1^NAc-VM^ projection increases dopamine release into NAc and promotes reward, whereas activation of D1^NAc-VP^ projection reduces dopamine level and elicits aversion. Activation of D1^NAc-VM^ and D1^NAc-VP^ projections drives opposing regulation of VM DAergic neurons and promotes opposite internal emotional states. Thus, we believe that D1-MSNs composed of different subpopulations within the NAc stringently control DA balance and responses to environmental stimuli.

As illustrated in Fig. 6l, we propose an updated working model of the regulation of mesolimbic D1-MSN circuits on dopamine release. D1^NAc-VM^ neurons, which express higher levels of D1 receptor, predominantly project to the VM and inhibit VM GABAergic neurons, disinhibiting DAergic neurons projecting back to the NAc and promoting dopamine release into the NAc. The functions of D1^NAc-VM^ neurons are consistent with the conventional role of D1-MSNs. Conversely, D1^NAc-VP^ neurons, which have lower levels of D1 receptor and predominantly target the VP to inhibit VP GABAergic neurons, ultimately disinhibit VM GABAergic neurons and thus inhibit the activity of VM DAergic neurons, hindering DA release into the NAc. Thus, the effects of activation of D1^NAc-VP^ and D1^NAc-VM^ pathways on dopamine release and emotional valence are opposing to each other. Moreover, activation of D1^NAc-VM^ neurons increases DA release and triggers the activation of D1^NAc-VP^, which inhibits DAergic neurons in the VM to reduce DA level in the NAc through net disinhibition of VM GABAergic neurons. This regulation of DA level in the NAc by D1^NAc-VP^ may occur when the DA level is higher than normal, since D1^NAc-VP^ neurons possess fewer D1 receptors than D1^NAc-VM^ neurons. VM GABAergic neurons receive direct inhibition from D1^NAc-VM^ neurons and indirect disinhibition from D1^NAc-VP^ neurons. These two neuronal populations cooperatively regulate the balance of NAc dopamine level and encode emotional valence through VM GABAergic neurons (Fig. 6l).

### D1^NAc-VM^ and D1^NAc-VP^ neuronal populations encode opposite valences

The prevailing hypothesis posits that D1-MSNs and D2-MSNs encode opposing valences. Activation of D1-MSNs induces reward and positive reinforcement, whereas activation of D2-MSNs produces aversion and negative reinforcement. However, some studies provide apparent contradictory results. Optical activation of D1-MSNs induces place reward or avoidance depending on their neuronal stimulation pattern,^38^ and optical stimulation of dynorphin-positive cells, which co-express D1 receptor, in dorsal and ventral NAc shell drives real-time place preference and avoidance respectively.^39^ A recent paper reported that VTA projecting D1-MSNs in the NAc medial and lateral shell have distinct effects on behavior. Specifically, the lateral projections to VTA produce place preference and ICSS, but the medial projections to VTA mostly suppress behavioral responding.^40^ Studies also show that both D1- and D2-MSNs are activated by aversive stimuli such as food restriction^41^ or footshock.^42^ In addition, one recent work shows that optogenetic activation of D2-MSNs in the dorsomedial NAcSh drives preference for stimulation side, whereas activation of ventral NAcSh D2-MSNs induces avoidance for stimulation side.^43^ These data suggest the diversity in valence encoding of MSNs in the NAc. In this study, we find that D1^NAc-VM^ neurons show properties that resemble those of the conventional D1-MSNs,^44^ promoting reward and positive reinforcement learning. By contrast, D1^NAc-VP^ neurons function opposite to the conventional D1-MSNs, eliciting aversion and negative reinforcement learning. These intermingled D1^NAc-VM^ and D1^NAc-VP^ neurons in the NAc showing functional diversity and opponency in valence production might attribute to the dichotomous results. Additionally, the opposing responses of D1^NAc-VM^ and D1^NAc-VP^ neurons to environmental stimuli might synergistically determine the valence state. The roles of heterogeneous NAc D1-MSNs in valence encoding deserve extensive study.

### D1^NAc-VP^ projection negatively regulates mesolimbic DA system

Previous studies suggest that the VP function is a major mechanism for reward in the brain. GABA_A_ receptor agonist muscimol injection in the VP attenuates saccharine flavored water intake and GABA_A_ receptor antagonist bicuculline increases eating behavior and food intake.^45,46^ The repeatedly pressing the level for electrical stimulation of the VP indicates that activation of the VP is sufficient to induce a reward.^47^ VP contains two major projection neuron types, including GABAergic neurons (GABA^VP^, 73% of all VP neurons) and glutamatergic (Glu^VP^, 23% of all VP neurons) neurons. Activation of GABA^VP^ neurons facilitates cocaine seeking during withdrawal of cocaine self-administration mice, but activation of Glu^VP^ neurons suppresses this effect.^48^ Activation of GABA^VP^ neurons drives place preference through the projection to the VM, while activation of Glu^VP^ neurons induces place avoidance through the projection to the lateral habenula.^49^ These studies suggest that activation of GABA^VP^ and Glu^VP^ neurons produces reward and aversion, respectively, through different downstream circuits. Moreover, the reward induced by activation of the VP should primarily be ascribed to activation of GABA^VP^ neurons, since Glu^VP^ neurons might function opposingly to GABA^VP^ neurons. Our retrograde tracing data show that the NAc sends more inputs onto GABA^VP^ neurons than Glu^VP^ neurons, and GABA^VP^ neurons send more projections to VM neurons than Glu^VP^ neurons do. Our photometry recording data reveal a negative regulation within the mesolimbic system that activation of NAc-VP projection (including D1^NAc-VP^ and D2^NAc-VP^) increases the activity of VM GABAergic neurons, then decreases the activity of VM DAergic neurons, and suppresses DA release in the NAc. Combined with our electrophysiological recording data and results from other groups that D1- and D2-MSNs from the NAc equally innervate the VP through the inhibitory projections,^10,50^ we speculate that the net effect of the inhibition of VM DAergic neurons and DA release in the NAc produced by stimulation of GABAergic D1^NAc-VP^ projection may primarily be ascribed to its inhibition onto GABA^VP^ neurons that disinhibit GABAergic neurons in the VM and finally suppress the activity of VM DAergic neurons. In addition, optical activation of D1^NAc-VP^ projection-induced negative valence could also be mediated by the GABAergic inactivation of the VP as suggested by studies mentioned above, and the data of inhibition of VM DAergic neurons obtained in this study.

It is speculated that activation of accumbal D1R by dopamine enhances excitability of D1-MSNs and activation of D2R decreases excitability of D2-MSNs, which may synergistically augment DA release, producing maximal rewarding effects. However, the suppression mechanism and the brake on DA release in the NAc are unclear. Our results of in vivo fiber photometry recording and behavioral tests indicate activation of D1^NAc-VP^ projection inhibits VM DAergic neurons, decreases DA level in the NAc, and induces aversion, opposite to the outcome of activation of D1^NAc-VM^ projection. The classical model points out that D1-MSNs in the NAc activate DAergic neurons through their inhibition of local VTA GABAergic neurons.^34,35^ Thus, our study provides the novel evidence that D1-MSNs can down-regulate DA release through the VP circuit. Fast increase of DA concentration resulting from phasic bursts in DA neurons may activate D1^NAc-VP^ neurons and further DA release is in turn suppressed through this negative regulation loop. Therefore, D1^NAc-VP^ MSN-mediated negative regulation of dopamine level is critical for dopamine homeostasis and reward-aversion state.

Cocaine, a psychoactive drug, attaches to the dopamine transporter and blocks the normal recycling process, resulting in a buildup of dopamine in the synapse. Our data show that the reward effects of cocaine might be canceled out by activation of D1^NAc-VP^ pathway that inhibits DA release in the NAc and results in an aversive state. Lithium, an antibipolar drug, is commonly used in the management of mood disorders along with drugs targeting central monoaminergic transmission.^51^ Studies show that administration of lithium salts inhibits locomotion induced by novel environment,^52^ suppresses DA-associated behaviors in experimental animal models,^53^ and decreases DA release.^54^ LiCl conditioning results in place avoidance^55^ and taste aversion.^56^ Although the effects of lithium on DA system are still unclear, our data reveal that the aversive effects of lithium might be mediated by either inhibition of D1^NAc-VM^ pathway or activation of D1^NAc-VP^ pathway through the suppression of DA release in the NAc. These data support that D1^NAc-VM^ and D1^NAc-VP^ pathways cooperatively regulate DA release into NAc and drive opposing emotional valence.

### Accumbal D1^NAc-VP^ and D1^NAc-VM^ MSNs possess dichotomous anatomical, electrophysiological, and molecular properties

In this study, we find that VP-projecting and VM-projecting D1-MSNs are largely two distinct populations, in contrast to the previous study that accumbens D1-MSNs largely collateralize to both the VP and VM.^57^ The reason for this discrepancy might be due to different strategies of retrograde labeling. Our results are consistent with one recent report suggesting that D1-MSNs in the NAc medial shell projecting to VM and VP are largely distinct populations that constitute multiple parallel output pathways with different circuit connectivity and cocaine induced synaptic plasticity.^58^

Several studies show that stimuli of positive and negative valence could both induce dopamine release in the NAc from the VM. Nature reward, such as sucrose consumption, evokes robust increase of fluorescence of DA sensor in both NAc core and shell, while aversive stimuli, such as unpredictable footshock and tail pinch, suppress fluorescence of DA sensor in the NAc core and the dmshell, but increase DA signal in the vmshell.^59,60^ A recent study using fibre photometry shows that footshock decreases the calcium fluorescence in VTA-NAc core and VTA-NAc dmshell DAergic projection, but increases the calcium fluorescence in VTA-NAc vmshell DAergic projections, while sucrose licking increases the calcium fluorescence in VTA-NAc shell DAergic projection.^28^ These studies suggest that positive stimuli increase DA release in the NAc core and shell, while negative stimuli decrease DA release in NAc core and dmshell, but increase DA release in the NAc vmshell. In this study, we implanted the fiber (400 μm) above the NAc core and dmshell, where DA release was recorded. Consistent with these studies, we find that positive stimuli increase, and negative stimuli decrease DA release in the NAc core and dmshell. VM dopamine neurons are heterogeneous in their anatomic and electrophysiological properties.^4,61^ The activities of D1^NAc-VP^ and D1^NAc-VM^ MSNs and their projections may be regulated by the different subpopulations of VM DAergic neurons that innervate different subregions of the NAc. Our smFISH data and single-cell sequence show that the expression level of D1 receptor in D1^NAc-VM^ -MSNs is significantly higher than that of D1^NAc-VP^ MSNs, suggesting that DA release induce different responses in D1^NAc-VP^ and D1^NAc-VM^ MSNs and the activation of D1^NAc-VP^ neurons may require greater DA release than D1^NAc-VM^ MSNs. In addition, GABAergic, glutamatergic innervation, and others peptide release might produce different influences on D1^NAc-VP^ and D1^NAc-VM^ MSNs as well.^62,63^

Our electrophysiological data showed lower mIPSC frequency and rheobase current in D1^NAc-VP^ neurons than in D1^NAc-VM^ neurons, suggesting less net inhibitory inputs onto D1^NAc-VP^ neurons with more depolarization and high excitability. The monosynaptic inputs to D1^NAc-VM^ and D1^NAc-VP^ neurons are different in the prefrontal cortex, amygdala, and thalamus. Thus, D1^NAc-VM^ and D1^NAc-VP^ neurons have distinct neural circuits and molecular profiling. The functional divergence between D1^NAc-VM^ and D1^NAc-VP^ neurons may be rooted in their different neural circuits and connectivity.

## Methods

### Animals

C57BL/6 male mice were purchased from the Shanghai Laboratory Animal Center, CAS. *D1-Cre* (#030989-UCD), *D2-Cre* (#032108-UCD), *D2-eGFP* (#036931-UCD) transgenic mice were purchased from The Mutant Mouse Resource and Research Center. *TH-Cre* (#008601), *Gad-Cre* (#010802), *vGlut2-Cre* mice (#016963), *D1-tdTomato* (#016204) were purchased from The Jackson Laboratory. Mice were group-housed and maintained on a 12 h light-dark cycle (i.e., light cycle; 8 am–8 pm) with food and water available ad libitum. 8–12 weeks old mice (25 ± 2 g) were used for behavioral experiments. All experiments were performed following the National Institutes of Health Guide for the Care and Use of Laboratory Animals and approved by the Animal Care and Use Committee of Shanghai Medical College of Fudan University.

### Preparation of adeno-associated viruses (AAVs)

The AAV preparation with a titer exceeding 2 × 10^12^ vector genome (vg) mL^−1^ was used. *AAV*_*9*_*-EF1α-DIO-GCaMP6s*, *AAV*_*9*_*-CAG-DIO-eGFP*, *AAV*_*9*_*-CAG-DIO-tdTomato*, *AAV*_*9*_*-EF1α-DIO-H2B-GFP*, *AAV*_*9*_*-EF1α-DIO-hChR2(H314R)-mCherry*, *AAV*_*9*_*-TH-FlpO, AAV*_*9*_*-EF1α-DIO-histone-eGFP-2a-TVA*, *AAV*_*9*_*-EF1α-DIO-RVG* and *RV-ENVA-deltaG-dsRed* (RV*d*G 5.0 × 10^8^ colony forming units (cfu)/mL) were generated and packaged by BrainVTA Co., Ltd. *AAV*_*9*_*-EF1α-DIO-mCherry*, *AAV*_*9*_*-EF1α-DIO-eNpHR3.0-eYFP*, *AAV*_*9*_*-EF1α-DIO-eYFP*, and *AAV*_*9*_*-EF1α-DIO-GCaMP6m* were generate and packaged by Neuron Biotech co., Ltd. *AAV*_*9*_*-hSyn-FLEX-Chrimson-tdTomato*, *AAV*_*2/retro*_*-CAG-FLEX-FlpO*, *AAV*_*2/retro*_*-hSyn-FlpO*, *AAV*_*2/retro*_*-hSyn-Cre, AAV*_*2/retro*_*-hSyn-eGFP, AAV*_*9*_*-hEF1a-fDIO-hM3D-mCherry*, *AAV*_*9*_*-hSyn-Con/Fon-EYFP*, *AAV*_*9*_*-hSyn-Con/Fon-hChR2(H134R)-EYFP*, *AAV*_*9*_*-EF1α-fDIO-hChR2(H314R)-mCherry, AAV*_*2/retro*_*-EF1α-DIO-EYFP*, and *AAV*_*2/retro*_*-EF1α-DIO-mCherry* were generated and packaged by Taitool Biological Co., Ltd.

### Stereotaxic surgery

Six-week-old mice were anesthetized with isoflurane (3.5% for induction, 1.5%–2% for maintenance), placed in a stereotaxic instrument (Stoelting Instruments), and injected with 150–200 nL AAV in the targeted brain regions with a 10-μL syringe and a 36-gauge blunt needle under the control of a UMP3 ultra micro pump (World Precision Instruments) at a rate of 50 nL/min. The needle was left for an additional 5 min before withdrawal. Mice were remained on a heating pad until fully recovered from anesthesia and given Baytril (10 mg per kg, subcutaneously) twice a day for 3 days. Mice were monitored daily and allowed to recover from surgery over 3 weeks before subsequent behavioral experiments.

For the tracer retrograde labeling of D1^NAc-VM^ and D1^NAc-VP^ neurons, CTB Alexa Fluor 647 (Thermo Fisher Scientific, Massachussetts, USA. 200 nL per side, 1 μg/μL) was injected into the VM (AP: −3.2 mm, ML: ± 0.4 mm, DV: −4.4 mm) and CTB488 was injected into the VP (AP: + 0.1 mm, ML: ± 1.2 mm, DV: −5.1 mm) of 8-week-old *D1-tdTomato* mice. CTB647 (200 nL per side, 1 μg/μL) was injected into the VM, and CTB555 was injected into the VP of 8-week-old *D2-eGFP* mice. Eleven days after surgery, mice were anesthetized with isoflurane and subject to intra-cardiac perfusion with 30 mL PBS followed by 50 mL of 4% paraformaldehyde (PFA). Coronal sections containing the NAc were sliced at 30 μm on a freezing microtome and mounted. Sections were imaged on a confocal (Olympus) with a 10× and 20× objective. Cell counting was performed manually with 10× images. For each mouse, two images per section were obtained at 4 different rostro-caudal coordinates of NAc, providing 8 images per NAc. Total cell counts of 8 images were calculated per mouse. Cell numbers and percentage values were averaged within a section for each mouse and then between mice for the final mean percentage value. Cell counting and colocalization analysis was performed manually with ImageJ (NIH).

For the retrograde labeling of GABA^VP-VM^ or vGlut2^VP-VM^ neurons, *AAV-DIO-H2B-eYFP* was injected into the VP and CTB555 (200 nL per side, 1 μg/μL) was injected into the VM of *Gad-Cre* or *vGlut2-Cre* mice. 4 weeks after injections, brains were perfused and 30 μm brain sections were sliced on a freezing microtome. Sections were imaged using a laser-scanning confocal microscope (Olympus) with a 10×, 20× objective, and 40× oil objective.

For ex vivo electrophysiology experiments, CTB488 or CTB555 (200 nL per side, 1 μg/μL) were injected bilaterally into the VP (AP: + 0.1 mm, ML: ± 1.2 mm, DV: −5.1 mm) or VM (AP: −3.2 mm, ML: ± 0.4 mm, DV: −4.4 mm) of *D1-tdTomato* or *D2-eGFP* mice. Electrophysiological experiments were conducted 10 days after CTB injection. In another cohort of mice, *AAV*_*2/retro*_*-CAG-FLEX-FlpO* was injected into the VM or VP and *AAV*_*9*_*-EF1α-fDIO-hChR2(H314R)-mCherry* was injected into the NAc of 6-week old *D1-Cre* mice. The functional connectivity of D1^NAc-VM^ and D1^NAc-VP^ projectors to the VP were assayed by recording light-evoked responses in the VP cells 4 weeks after AAV injection.

For rabies virus-based monosynaptic tracing, 100 nL of a 1:1 volume mixture of *AAV*_*9*_*-EF1α-DIO-his-EGFP-2a-TVA* and *AAV*_*9*_*-EF1α-DIO-RVG* was injected into the NAc of *D1-Cre* mice or the VP of *Gad-Cre* and *vGlut2-Cre* mice. Two weeks later, *RV-ENVA-deltaG-dsRed* was injected into the VP or VM. Then rabies injection mice were housed in P2 lab for 8 days to allow for RV*d*G spread and dsRed expression. The counting of inputs was conducted blind to the experimental group.

For optical stimulation, *AAV*_*9*_*-EF1α-DIO-hChR2(H314R)-mCherry*, *AAV*_*9*_*-EF1α-DIO-eNpHR3.0-eYFP*, or the control virus was injected into the NAc (AP: + 1.8 mm, ML: ± 0.8 mm, DV: −4.6 mm), and optical fibers (200 μm diameter, 0.37 numerical aperture (NA), Hangzhou Newdoon Technology) were implanted bilaterally above the VP (AP: + 0.1 mm, ML: ± 2.0 mm, DV: −5.1 mm, 10° angle) or VM (AP: −3.2 mm, ML: ± 1.2 mm, DV: −4.5 mm, 10° angle). Fibers were stabilized in place using dental cement.

For the in vivo photometry recording, we recorded calcium fluorescence of D1-MSN terminals in the VM or VP during presenting the rewarding or aversive stimulus in the freely moving mice. *AAV*_*9*_*- EF1α-DIO-GCaMP6m* was injected into the NAc (AP: + 1.8 mm, ML: ± 0.8 mm, DV: −4.6 mm), one optical fiber (400 μm diameter, 0.48 NA, Hangzhou Newdoon Technology) was implanted into the VM or VP.

To record calcium fluorescence of VM GABAergic or DAergic neurons by stimulation of D1^NAc-VM^ projection, *AAV*_*9*_*-hSyn-FLEX-Chrimson-tdTomato* was injected into the NAc (AP: + 1.8 mm, ML: ± 0.8 mm, DV: −4.6 mm). Then *AAV*_*9*_*-EF1α-DIO-GCaMP6s* were injected into the VM (AP: −3.2 mm, ML: ± 0.4 mm, DV: −4.4 mm) of *Gad-Cre* mice, or *AAV*_*9*_*-EF1α-fDIO-GCaMP6s* and *AAV*_*9*_*-TH-FlpO* were injected into the VM of *D1-Cre* mice. Two optic fibers were implanted over the VM. One fiber for optical stimulation (200 μm diameter, 0.37 NA, Hangzhou Newdoon Technology) was implanted in the VM with a 10° angle (AP: −3.2 mm, ML: ± 1.2 mm, DV: −4.5 mm). Another fiber (400 μm diameter, 0.48 NA, Hangzhou Newdoon Technology) for photometry recording was implanted in the VM (AP: −3.2 mm, ML: ± 0.4 mm, DV: −4.3 mm). This allows us to shine yellow light (594 nm) into the VM through one optic fiber to activate D1-MSN GABAergic axon terminals arising from the NAc expressing ChrimsonR, while shining low levels of blue light (473 nm) through the second optic fiber to excite GCaMP6s expressing VM GABAergic or DAergic neurons, and measure emitted green (525 nm) fluorescence using fiber photometry.^64,65^

To record calcium fluorescence of VM GABAegic and DAergic neurons by stimulation of D1^NAc-VP^ projection, *AAV*_*9*_*-hSyn-DIO-ChR2-mCherry* was injected into the NAc. Then *AAV*_*9*_*-EF1α-DIO-GCaMP6s* were injected into the VM (AP: −3.2 mm, ML: ± 0.4 mm, DV: −4.4 mm) of *Gad-Cre* mice, or *AAV*_*9*_*-EF1α-fDIO-GCaMP6s* and *AAV*_*9*_*-TH-FlpO* were injected into the VM of *D1-Cre* mice. One optic fiber for optical stimulation was implanted in the VP (AP: + 0.1 mm, ML: ± 1.2 mm, DV: −5.1 mm), and another optic fiber for photometry recording was implanted over the VM.

### Ex vivo electrophysiology

#### Brain tissue preparation

Living acute brain slice preparation for electrophysiological recording was performed as previously described.^66^ Mice were anaesthetized with isoflurane (3.5% induction, 1.5%–2% maintenance) and perfused transcardially with 20 ml ice-cold and oxygenated (95% O_2_, 5% CO_2_) cutting solution containing (in mM): 93.0 NMDG, 93.0 HCl, 2.5 KCl, 1.2 NaH_2_PO_4_, 30.0 NaHCO_3_, 20.0 HEPES, 25.0 Glucose, 5.0 Sodium ascorbate, 2.0 Thiourea, 3.0 Sodium pyruvate, 10.0 MgSO_4_, 0.5 CaCl_2_ (pH 7.3–7.4, 295–305 mOsm). The brains were rapidly removed and placed in ice-cold and oxygenated cutting solution. The coronal slices (300 μm) containing the NAc were prepared with a semiautomatic vibrating blade microtome (HM760V, Thermo) and then transferred to the incubation chamber at 32 °C with the oxygenated cutting solution for 12 min. After the initial recovery period, the slices were kept in the modified aCSF containing (in mM): 94.0 NaCl, 2.5 KCl, 1.2 NaH_2_PO_4_, 30.0 NaHCO_3_, 20.0 HEPES, 25.0 Glucose, 5.0 Sodium ascorbate, 2.0 Thiourea, 3.0 Sodium pyruvate, 2.0 MgSO_4_, 2.0 CaCl_2_ (pH 7.3–7.4, 295–305 mOsm) at room temperature under constant carbogenation. For whole-cell patch-clamp recording, the slices were transferred to the recording chamber perfused with 32 °C carbogenated recording aCSF containing (in mM): 124.0 NaCl, 2.5 KCl, 1.2 NaH_2_PO_4_, 30.0 NaHCO_3_, 20.0 HEPES, 25.0 Glucose, 5.0 sodium ascorbate, 2.0 Thiourea, 3.0 sodium pyruvate, 2.0 MgSO_4_, 2.0 CaCl_2_ (pH 7.3–7.4, 295–305 mOsm) at a rate of 1.5 mL/min.

#### Measurements of intrinsic membrane properties

The recording electrodes (4–6 MΩ resistance) were filled with an internal solution containing (in mM) 120.0 K-gluconate, 5.0 NaCl, 1.0 MgCl2, 0.2 EGTA, 10.0 HEPES, 2.0 MgATP, and 0.1 NaGTP (adjusted to pH 7.2 with KOH, 280–290 mOsm). After recording the resting membrane potential of each neuron, the slow polarizing current was applied to hold the neurons at −70 mV. To measure the firing frequency of action potentials, depolarizing current steps (from 0 to 200 pA in 20 pA increment, 1-s duration per each step) were applied. After completion of the current step, an additional depolarizing current step (in 5 pA unit increment) was injected again to find rheobase.^67,68^ The intrinsic membrane properties were analyzed with Clampfit (Clampfit 10.2). *Spontaneous EPSCs and IPSCs*. For recording miniature excitatory postsynaptic currents (mEPSCs) and miniature inhibitory postsynaptic currents (mIPSCs), patch electrodes were filled with an internal solution containing (in mM) 120.0 CsMeSO_4_, 15.0 CsCl, 10.0 TEA-Cl, 8.0 NaCl, 10.0 HEPES, 0.2 EGTA, 4.0 MgATP, 0.3 NaGTP, and 5.0 QX-314 (pH 7.3, 280–290 mOsm). mEPSCs (> 300 events per cell) were collected at −70 mV in the presence of TTX (Tetrodotoxin, 1 µM), picrotoxin (100 μM), and D-AP5 (D-2-amino-5-phosphono-valeric acid, 50 µM). mIPSCs were collected at 0 mV in the presence of TTX (1 µM), D-AP5 (50 µM) and NBQX (2,3-dihydroxy-6-nitro-7-sulfamoyl-benzo(f)quinoxaline, 100 µM). The signals were acquired at 20 kHz and filtered at 2 kHz. The series-resistance was < 20 MOhm. mEPSCs and mIPSC were analyzed with Minianalysis (Synaptosoft).^69,70^

#### PPRs

The internal solution containing (in mM) 120 KCl, 30 potassium gluconate, 4.0 MgCl_2_, 10 sodium creatine phosphate, 1.1 EGTA, 5 HEPES, 3.4 NaATP, and 0.1 NaGTP (adjusted to pH 7.2 with KOH, 280–290 mOsm). Light-evoked IPSCs were evoked by 4-ms-long LED pulse that was transmitted on the slice through the light path of the microscope and recorded in the presence of TTX (1 µM), D-AP5 (50 µM), and NBQX (100 µM) at −70 mV in voltage-clamp model. PPRs were obtained 5 min after invading the cell at an interval of 50 ms for 30 consecutive traces. The PPR was calculated as the peak amplitude ratio of the second to the first IPSC.^10,50^

#### Photostimulation of brain slices

To test spike fidelity of optogenetic stimulation of ChR2 and inhibitory effects of eNphR3.0, brain slices containing the NAc were chosen for current-clamp recording with the internal solution containing (mM): 120.0 K-gluconate, 5.0 NaCl,1 MgCl_2_, 0.2 EGTA, 10.0 HEPES, 2.0 MgATP, and 0.1 NaGTP (adjusted to pH 7.2 with KOH, 280–290 mOsm). Blue light (473 nm; duration 5 ms; intensity ~3 mW) was delivered at the frequency of 5 Hz, 10 Hz, 20 Hz to trigger the action potential. Yellow light (594 nm, intensity ~3 mW) was delivered to eliminate the action potential induced by 100 pA current stimulation. To measure the function and specificity of CNO stimulation of hM3Dq in D1-MSNs, brain slices containing the NAc were chosen for current-clamp recording. Rheobase was defined as the minimal current amplitude required for firing an action potential with a depolarizing current step (in 5 pA unit increment) and measured before and 15 min after CNO application to the bath solution (5 µM).

During the execution and analysis of the electrophysiological recordings, the experimenter was blind to the genotypes of the individual animals. The signals were acquired at 20 kHz and filtered at 2 kHz. The series-resistance was < 20 MOhm. Data with series resistance changed by > 20% were excluded. mEPSCs and mIPSCs were analyzed using Minianalysis. The spike frequency, rheobase, and PPRs were analyzed using Clampfit.

### Behavioral scheme

#### Conditioned place preference/avoidance (CPP/CPA)

CPP/CPA was performed as previously reported.^71^ The optical fiber was connected to a fiberoptic cable and mice were placed in a rectangular apparatus consisting of left and right chambers (15 cm × 15 cm each). One chamber had black and white striped walls and frosted floor and the other had black and white checkered walls and black floor. Before behavioral sessions, mice were gently attached to a fiberoptic patch cord with optical fiber via a ceramic sleeve (Hangzhou Newdoon Technology). The patch cable was also connected to a fiberoptic rotary joint (Doric Lenses), which permitted free rotation while transmitting light from an upstream 473/594 nm DPSS laser diode (Shanghai Dream Lasers Technology Co., Ltd.), and laser output was controlled using a mini-Master pulse stimulator (Thinker Teck Nanjing Biotech). Power output was tested at the tip of optic fiber and was checked before and after each experimental animal. On day 1, mice were allowed to explore the entire apparatus for 15 min. On days 2–4, mice were confined to one of the chambers paired with optical stimulation (ChR2: 473 nm, 10 mW, a train of twenty 5-ms light pulses at 20 Hz every 10 s; eNpHR3.0: 594 nm, 5 mW, 3-min epochs with 3-min intervals for 30-min) in the morning and confined in the other chamber for 30 min without optical stimulation in the afternoon. Mice were presented with two conditioning trials for 30 min separated by 6 h each day. The laser or no laser paired conditioning was performed alternatively in the morning or afternoon and repeated for 3 days. On day 5 mice were allowed to freely explore the entire apparatus for 15 min. A video camera positioned above the chamber recorded the trial, and mouse locations were tracked and analyzed using Ethovision XT software (Noldus, Wageningen, Netherlands). CPP scores were calculated by subtracting the time spent in the non-stimulated side from the time spent in the stimulated side.

To investigate the effects of activation or suppression of D1^NAc-VM^ and D1^NAc-VP^ projections on cocaine-CPP and LiCl-CPA, optical fibers were connected to the cannula before injection of saline or cocaine (10 mg/kg) or LiCl (150 mg/kg) and perform the optical stimulation during cocaine or LiCl conditioning with the protocol as described as above. No laser stimulation was presented during saline conditioning. Cocaine/LiCl and saline paired conditioning was performed alternatively in the morning or afternoon and repeated for 3 days. On day 5 mice were allowed to freely explore the entire apparatus for 15 min. A video camera positioned above the chamber recorded the trial, and mouse locations were tracked and analyzed using Ethovision XT software (Noldus, Wageningen, Netherlands).

The rewarding or aversive magnitude of optical activation of D1^NAc-VM^ and D1^NAc-VP^ projections was examined with cocaine CPP or LiCl CPA paradigm. The mice were conditioned with saline injection on one side and cocaine injection on the other side of the chamber. Optical activation of D1^NAc-VM^ projection was presented during saline conditioning. Another cohort of mice was conditioned with saline injection on one side and LiCl injection on the other side. Optical activation of D1^NAc-VP^ projection was presented during saline conditioning. Cocaine/LiCl and saline paired conditioning was performed alternatively in the morning or afternoon and repeated for 3 days. One day later the mice were allowed to freely explore the entire apparatus for 15 min.

#### Real-time place preference/avoidance testing (RTPP/A)

Mice were placed in a rectangular apparatus consisting of left and right chambers (15 cm × 15 cm each). Mice were allowed free moving between compartments for 20 min. Each entry into the same chamber was paired with optical stimulation (ChR2: 473 nm, 10 mW, 5 ms pulses at 20 Hz upon entry). A video camera positioned above the chamber recorded each trial and mouse locations were tracked and analyzed by Ethovision XT software (Noldus, Wageningen, Netherlands). Difference scores were calculated by subtracting the time spent in the non-stimulated chamber from the time spent in the stimulated chamber.

#### ICSS

ICSS was performed as previously reported.^40,72^ Mice were placed into soundproofing operant chambers (24 cm  × 20 cm × 18 cm, L × W × H, Shanghai VanBi Intelligent Technology Co., Ltd.) containing two illuminated nose-poke ports (“active” and “inactive”). A nose-poke response to the active port was accompanied by illumination of cue-light and delivery of optical stimulation (ChR2: 473 nm, 10 mW, 5 ms pulses at 20 Hz, 1 s duration), while a nose-poke response in the “inactive” port resulted in no optical stimulation or cue-light delivery. Light stimulation was controlled by a computer running Tracking Master V3.0 software (Shanghai VanBi Intelligent Technology Co., Ltd.), which recorded times of nosepoke.

#### Intra-cranial light administration in specific subarea (ICLASS)

ICLASS was performed as previously reported.^73^ An illuminated open field (40 cm × 40 cm, L × W) was used for this behavioral experiment. Within the open field, red lines marked a central area (20 cm × 20 cm, L × W). Before behavioral sessions, mice were gently attached to a fiber optic patch cord with optical fiber via a ceramic sleeve (Hangzhou Newdoon Technology). Optical stimulation was controlled by a computer running Ethovision XT software (Noldus, Wageningen, Netherlands), which videotaped and analyzed location and movements in the central and the periphery arena. The ICLASS task began 3 weeks after AAV injection. The mouse was gently released from the center of the open field and allowed for exploration for 15 min, and the locomotion was monitored and calculated. Whenever the centroid of the mouse body was located within the central or the periphery arena, blue light pulses were passed to the D1^NAc-VM^ and D1^NAc-VP^ projections through the optical fiber (ChR2: 473 nm, 10 mW, 5 ms pulse at 20 Hz upon entry). Mouse movements and the percent ratio of the time spent in the central and the periphery arena were tracked and analyzed by Ethovision XT software (Noldus, Wageningen, Netherlands).

### Fiber photometry recording

#### Response to sucrose licking

Delivery of water was controlled by a microcontroller-based behavioral system running on Tracking Master V3.0 software (Shanghai VanBi Intelligent Technology Co., Ltd.). Licks were detected by a custom-made lickometer with a capacitive touch sensor and a microcontroller. Fiber photometry was performed as previously reported.^47^ Before recording day, mice were water-deprived for 12 h, Then they were introduced to sucrose licking chamber. 20 μL water was delivered by the lick of the water spout followed with a 10-s timeout. The onset of each licking behavior was tagged by triggering TTL signal (Shanghai VanBi Intelligent Technology Co., Ltd.), which was synchronously output to the fiber photometry system. The TTL signal and fluorescence signal were recorded simultaneously by a fiber photometry system (Thinker Tech Nanjing Biotech). Mouse behaviors and fluorescence signals were captured simultaneously. Fluorescence signals during the first five times of licking behavior of each mouse were analyzed.

#### Response to retreat behavior

Mice were habituated to a rectangular open-field with a video capture system (40 × 40 × 40 cm^3^, L × W × H, Shanghai VanBi Intelligent Technology Co., Ltd.) 30 min per day for 3 days. After this habituation session, mice were exposed to the chamber for 10 min, then a novel object was introduced into the center. Mouse behaviors and fluorescence signals were captured simultaneously. The first three retreat behaviors were manually tagged through videos by researchers blind to the experiment groups and averaged to obtain a time-locked response.^21^

#### Response to sucrose pellet consumption

Mice with free access to food and water were subjected to photometry recording during consumption of sucrose pellet (Bio-Serv, Co., Flemington, NJ, USA, F07595). The mice were habituated to a rectangular chamber with a video capture system (20 × 10 × 20 cm^3^, L × W × H, Shanghai VanBi Intelligent Technology Co., Ltd.) 30 min per day for 3 days. A dish of sucrose pellets (MLabRodent Tablet 20MG; TestDiet) was introduced into one corner of the chamber. Then the mice were introduced to the chamber. Mouse behaviors and fluorescence signals were captured simultaneously. Fluorescence signals during the first three to five times of consumption behavior of each mouse were analyzed.

#### Response to social interaction

The mice were habituated to a rectangular open-field with a video capture system (40 × 40 × 40 cm^3^, L × W × H, Shanghai VanBi Intelligent Technology Co., Ltd.) 30 min per day for 3 days. After the habituation session, mice were exposed to the open-field for 10 min, then a female stranger mouse (6 weeks old) was introduced into the open-field. The social behaviors and fluorescence signals were captured simultaneously. Sniffing female refers to the time in which the male’s nose is coming closely toward the female’s face and/or body. Fluorescence signals during sniffing the female mouse were analyzed.

#### Response to unexpected air puff

Animals were introduced into a previously familiar chamber (40 × 40 × 40 cm^3^, L × W × H). In a single experimental session, ten unexpected air puffs were randomly delivered to the eye of mice with inter-trial intervals of 1 min to 5 min. All the mice underwent sucrose licking trials, the retreat behavior next, and then the unexpected air puff to minimize the possible effect of the previous stimulus on subsequent experiments. Mouse behaviors and fluorescence signals were captured simultaneously. The onset of each air puff was recorded by the performer and manually tagged through videos by students blind to the experimental groups. Fluorescence signals during the first five times of air puff of each mouse were analyzed.

#### Response to tail suspension

Animals were introduced into the same chamber (40 × 40 × 40 cm^3^, L × W × H). After sucrose consumption trials, the retreat behavior next, and then the unexpected air puff, tail suspension was delivered 10 times with inter-trial intervals of 1 min to 5 min. The tail of mice was chased and grabbed by hand. We grabbed and suspended the tail of the tested mice fast to reduce the interference of irrelevant stimuli. The height of tail suspension was about 40 cm height from the bottom of the chamber. After grabbing, the mice were suspended in air for 7 s or 10 s before releasing them in the chamber. Mouse behaviors and fluorescence signals were captured simultaneously. The onset of each tail suspension was recorded by the performer and manually tagged through videos.

#### Response to cocaine conditioning, cocaine CPP test, and optical stimulation induce CPP/A test

Animals were acclimated to the behavior room before conditioning. A 1-m-long fiber-optic patch cord (Doric Lenses) was connected to the implanted optical fiber targeting the VM or VP with a zirconia sheath and was suspended above the experimental environment to allow animals to move freely. In cocaine-CPP task, fluorescence signals were captured during the first pair of saline and cocaine conditioning, and CPP test. In optical stimulation induce CPP/A task, fluorescence signals were captured during CPP/A test. The signals during conditioning and entrances to the cocaine or stimulation paired side were analyzed.

#### Photometry recording for DA release

Mice were placed into a chamber with a patch cord and subjected to sucrose consumption, retreat behavior, unexpected air puff, and tail suspension for recording DA-sensor signals. The stimuli were delivered as mentioned above. For stimulation of D1-MSN or D2-MSN terminals in the VM or VP, optical stimulation (473 nm, 10 mW, 5 ms pulse at 20 Hz, 1 s duration) was given every 10–30 s for 10 trials through one optic fiber implanted in the VM or VP. The other fiber planted in the NAc was used for both exciting and recording from the genetically encoded indicator of DA-sensor (DA2m) in real-time.

#### Fiber photometry analysis

The laser power was adjusted at the tip of optical fiber to the low level of 10–20 μW, to minimize bleaching. The GCaMP or DA-sensor signal was collected and converted to voltage signals. The analog voltage signals were digitalized at 100 Hz and recorded by a fiber photometry system developed by Dr. Luo’s lab^74,75^ (Thinker Tech Nanjing Biotech). The data were segmented based on behavioral events within individual trials. For recording the response of axon-GCaMP and DA2m to the stimuli in free-moving mice, fluorescence values were obtained before the stimuli and during sucrose licking, sucrose pellet consumption, sniffing of the female stranger, retreat from the novel object in the center, airpuff (0–5 s) and tail suspension (0–10 s). The data were segmented based on behavioral events within individual trials. ΔF/F of the 2-s before stimulation were taken as the baseline. The in vivo fiber photometry recordings were analyzed by the students blind to the experimental group. Photometry data were analyzed with custom-written MATLAB codes (MATLAB R2019b, MathWorks). The frequency of Ca^2+^ transient events in D1^NAc-VM^ and D1^NAc-VP^ projectors during cocaine conditioning was analyzed as the previous study.^76^ In brief, fluorescence signals were normalized (ΔF/F) during a 20 s (±10 s) window around each data point. A value of 2.91 median absolute deviations of baseline before drug treatment was used as a threshold for detecting events during 5–10 min after saline or cocaine injection during conditioning.

### Single-cell RNA sequencing

#### Neuronal isolation of the fluorescence-labeled cell

C57 mice (8 weeks) were injected with CTB555 in the VP or VM. Eleven days after CTB labeling, mice were processed to a single cell suspension as the previous research.^77^ Briefly, mice were anesthetized with isofluorane (3.5% for induction, 1.5–2% for maintenance) and then decapitated. The brain was quickly removed and transferred to cold ACSF bubbled with carbogen (95% O_2_/5% CO_2_) for 5 min before being sectioned into 300 μm sections with a vibratome. NAc area was collected and digested in Hibernate-A medium containing 10 U/mL Papain and 100 U/mL DNAse I at 28 °C for 5 min with gentle trituration. The suspension was then filtered with a 40 μm mesh and purified with percoll gradient. Then the cells were resuspended in Hibernate-A medium with 0.04% BSA and kept on ice. The CTB555^+^ cells were picked up manually under a fluorescence microscope. Bilateral NAc from five mice each group was used for this experiment.

#### Single-cell RNA-seq library preparation

Single-cell transcriptional profiling was performed following the methods from Tang et al. ^78^ Single cells were picked into the cell lysis buffer containing barcoded reverse transcription primers by mouth pipette. Reverse transcription and amplification were performed as described for Smartseq2, except that a second-round amplification was performed using 3' biotinated primer. Then the PCR products with different barcodes were pooled together, sonic disrupted into ~300 bp fragments, 3' enriched, and then used for library construction. The libraries were processed on the Illumina platform for sequencing of 150 bp pair-end reads, approximatively 500 M raw data were acquired for each cell.

By retrograde tracing as mentioned above, we manually isolated retrograde-labeled NAc cells projecting from VP and VM pathways under fluorescence microscope, and performed single-cell transcriptome sequencing. In three independent experiments, 478 cells from VP group (NAc → VP), and 214 cells from VM group (NAc → VP) were collected. We assessed cDNA size (Supplementary information, Fig. S7a), sequencing saturation status (Supplementary information, Fig. S7b), genes detected (Supplementary information, Fig. S7c) to evaluate library quality, which all met conventional Smart-seq2 standards.^79^ Data from Ho et al. and Gokce et al. were also included to aid data quality evaluation and cell-type clustering. From the 692 cells analyzed, 562 were neurons, characterized by comparing with annotated data from Gokce et al. Then we utilized striatal single-cell data from Ho et al., in which D1 and D2-positive cells from transgenic mice were manually isolated and sequenced, to see if there was spatial separation of D1 and D2 cells based on UMAP (Uniform Manifold Approximation and Projection) clustering. As there was no clear segregation of these MSNs, we simply picked out 209 Drd1-expressing cells for further analysis, in which 131 cells were NAc→VP, and 78 were NAc→VM.

#### Single-cell RNA-seq analysis

Raw sequences were demultiplexed using zUMI software^80^ and then aligned to the Ensembl mouse genome (GRCm38) with STAR (version 2.7.0a).^81^ Raw count for each gene was used for subsequent major cell type determination using Seurat,^82^ and DESeq2^83^ was used for within-cluster differential gene expression analysis. ClusterProfiler^84^ was used to analyze gene ontology enrichment.

### Immunofluorescence

Mice were transcardially perfused with 0.9% saline followed by 4% PFA (dissolved in 0.1 M Na_2_HPO_4_ / NaH_2_PO_4_ buffer, pH 7.5). Brains were post-fixed in 4% PFA at 4 °C for 4 h and then transferred to 30% sucrose/PBS solution for 3 days. Then brains were sectioned into 30-μm-thick slices, which were then stored in the cryoprotective buffer at −20 °C. For immunostaining, each slice was placed in PBS and washed three times in PBS, followed by incubation with primary antibody (anti-TH,^85^ rabbit, 1:1,000, Millipore AB152) at 4 °C overnight. After being rinsed in with PBS, the brain slices were incubated with fluorescence conjugated secondary antibody Alexa-647 (rabbit, 1:50000, Jackson ImmunoResearch) at room temperature for 2 h. Finally, slices were coverslipped with the anti-quenching mounting medium (Thermo Fisher Scientific).

### FISH by RNAscope

Three weeks after injection of *AAV*_*2/retro*_*-hSyn-eGFP* in the VM or VP of C57 mice, the mice were perfused intracardiacally with saline first, then with 4% PFA in 0.1 M Na_2_HPO_4_/NaH_2_PO_4_ buffer (pH 7.5) and the brains were removed. After post-fixation in 4% paraformaldehyde for 4 h, the samples were stored in 30% sucrose/PBS for 3 days. FISH was performed on the fixed frozen brain slices containing the NAc with 10-μm thick slices, following the RNAscope procedures (Advanced Cell Diagnostics, Inc., Newark, CA, USA). In brief, frozen sections were cut coronally through the NAc formation. Sections were thaw-mounted onto Superfrost Plus Microscope Slides (Fisher Scientific, Waltham, USA) and pretreated for protease digestion for 10 min at room temperature. Sections from *AAV*_*2/retro*_*-hSyn-eGFP* injection mice were then incubated with probes of mouse *Drd1* and *Markers* for 2 h at 40 °C with labeled probe mixture per slide (*Drd1*, accession No: NM_010076.3, target region 444-1358; *Drd2*, accession No: NM_010077.2, target region 69-1175; *Markers*: *Tac1*, accession No: NM_009311.2, target region 20-1034; *Pdyn*, accession No: NM_018863.3, target region 33-700; *Chrm4*, accession No: NM_007699.2, target region 400-1330; *Isl1*, accession No: NM_021459.4, target region 145-1437; *Slc35d3*, accession No: NM_029529.3, target region 455-1603; *Penk*, accession No: NM_001002927.2, target region 106-1332; *Adk*, accession No: NM_134079.4, target region 152-1137, *Adora2a*, accession No: NM_009630.2, target region 152-1222; *Gpr6*, accession No: NM_199058.1, target region 138-1027; *Gpr52*, accession No: NM_001146330.1, target region 4-981; *Foxp1* accession No: NM_053202.2, target region 1101-2194; *Htr7*, accession No: NM_008315.2, target region 1516-2490; *Sp9*, accession No: NM_001005343.2, target region 2-960; *Arc*, accession No: NM_018790.2, target region 23-1066; *EYFP*, accession No: KF450806.1, target region 7768-8420; *mCherry*, accession No: MH492388.1, target region 23-681). The nonspecifically hybridized probe was removed by washing the sections in 1× washing buffer at room temperature, followed by Amplifier 1-FL for 30 min, Amplifier 2-FL for 30 min and Amplifier 3-FL for 15 min at 40 °C. Each amplifier was removed by washing with 1× washing buffer for 2 min at room temperature. At least six brain slices from each mouse were performed RNAscope and imaged.

### Confocal microscopy and image analysis

Confocal fluorescence images were acquired with Nikon A1 confocal laser scanning microscope using a 10× or 20× objective for imaging stained or autofluorescent neurons. The center of the viral infection was taken at the brightest fluorescent point. The tip of the fiber or cannulas was determined by the ~50 μm thick gliosis generated by the fiber.

Cell counting was conducted using Image J. Brain regions were defined regarding the Allen Mouse Brain Reference Atlas, the areas were quantified by applying the scale calibration. FISH images of co-localization of eGFP*/Drd1/Marker* cells were determined using confocal images of the NAc in 6 slices per brain. The number of triple-positive cells was divided by the number of double-positive cells (eGFP*/Drd1*^+^) as the expression levels in D1^NAc-VP^ and D1^NAc-VM^ MSNs. The counting was done by an experimenter blind to the groups.

FISH images of *Drd1* expression in the D1^NAc-VP^ and D1^NAc-VM^ neurons were analyzed using custom MATLAB as previously described.^86^ Briefly, six slices from each mouse containing NAc were used. DAPI staining was used to localize cell bodies. Puncta of FISH molecules were counted within *Drd1* and eGFP double-positive cells. FISH images of *Arc and Klf5* expression in the D1^NAc-VP^ (*EYFP*^+^) and D1^NAc-VM^ (*mCherry*^+^) neurons were also analyzed by smFISH. Puncta of Arc of Klf5 were counted within *EYFP*^+^ or *mCherry*^+^ cells.

### Quantification and statistical analyses

Experimental data were presented as means ± SEM, analyzed by SPSS and MATLAB, and plotted by Graphpad Prism. Single-variable comparisons between two groups were analyzed with two-tailed Student’s *t*-test. Multiple group comparisons were analyzed using one-way or two-way Analysis of Variance (ANOVA), followed by *Bonferroni’s* post hoc test. In detail, group differences of behavioral tests were detected using repeated measures (RM) ANOVA, followed by *Bonferroni’s* post hoc tests with sessions as a within-subjects factor and CNO treatment or optical stimulation as a between-subjects factor. The electrophysiological data were tested for significance using two-tailed Student’s *t*-test, or RM ANOVA, followed by *Bonferroni’s* post hoc tests with current injection as a within-subjects factor and neuronal type as a between-subjects factor. Photometry data were analyzed with two-tailed Student’s paired *t*-test for fluorescence of ΔF/F. Immunofluorescence data were analyzed by two-tailed Student’s *t*-test or one-way ANOVA. FISH data were analyzed by two-tailed Student’s *t*-test. The non-normalized data were analyzed with Mann-Whitney U test, Kruskal-Wallis one-way ANOVA on Ranks, or RM ANOVA with Geisser-Greenhouse correction. Full statistical analyses corresponding to each data set are presented in Supplementary information, Table S1.

## Supplementary information

Supplementary Figure 1

Supplementary Figure 2

Supplementary Figure 3

Supplementary Figure 4

Supplementary Figure 5

Supplementary Figure 6

Supplementary Figure 7

Supplementary Figure 8

Supplementary Figure 9

Supplementary Figure 10

Supplementary Table S1

## Data Availability

All data are available from the corresponding author upon reasonable request. Single-cell RNA sequencing data have been deposited in the Gene Expression Omnibus under accession number PRJNA692326.

## Code availability

Custom MATLAB codes are available from the corresponding author upon reasonable request.
