# Supplementary material for: A distinct D1-MSN subpopulation down-regulates dopamine to promote negative emotional state
Source: Cell Res. 2021 Nov 30;32(2):139–56. doi: 10.1038/s41422-021-00588-5 (PMC8807621; doi:10.1038/s41422-021-00588-5)
Supplement: Supplementary file 1 — Supplementary Figure 1 [file 41422_2021_588_MOESM1_ESM.pdf]

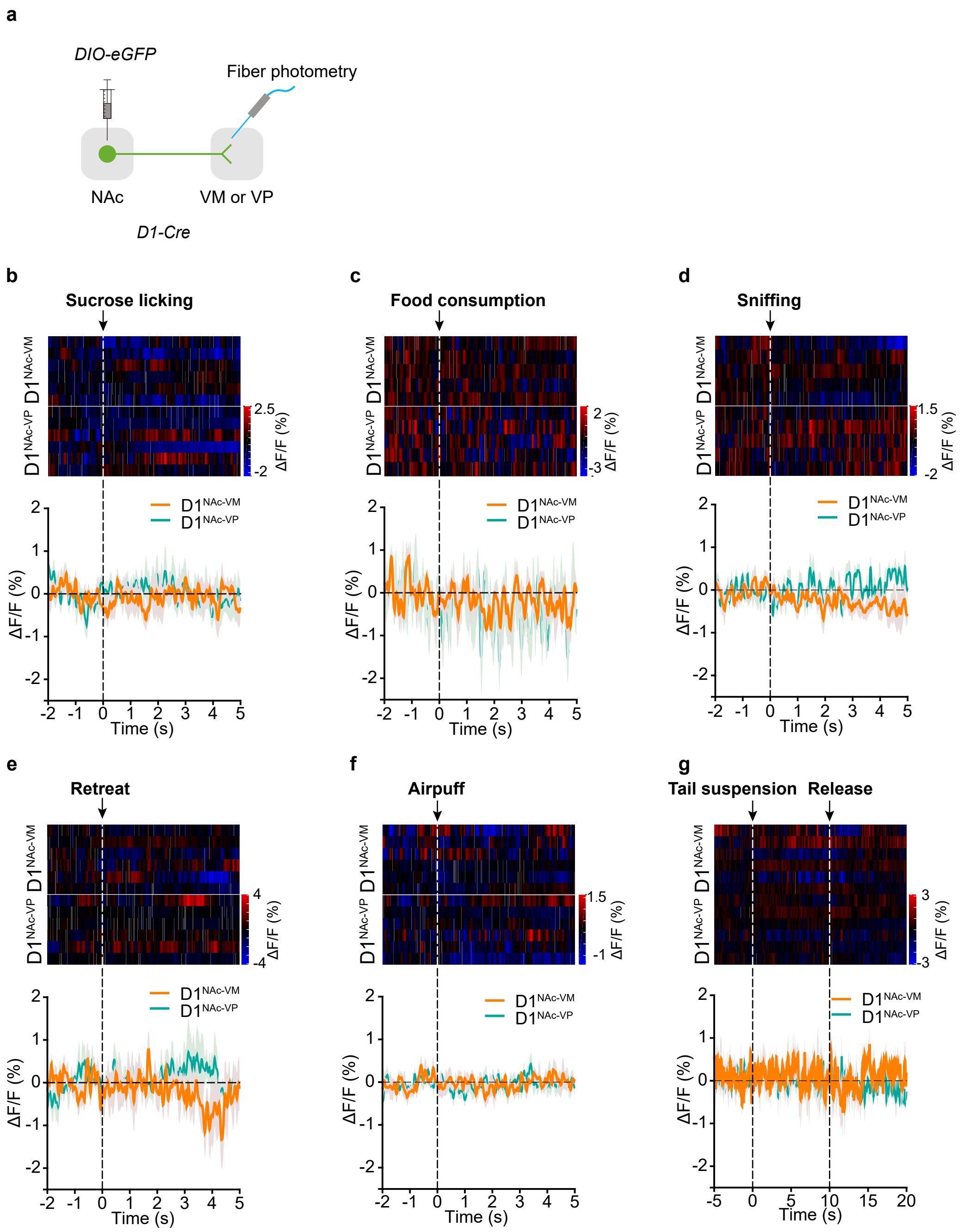

a
DIO-eGFP
Fiber photometry
NAc
VM or VP
D1-Cre
b
Sucrose licking
c
Food consumption
d
Sniffing
e
Retreat
f
Airpuff
g
Tail suspension
Release
D1NAc-VM
D1NAc-VP
ΔF/F (%)
Time (s)

Supplementary information, Fig. 1 The changes of GCaMP6m signal in D1$^{NAc\text{-}VM}$ and D1$^{NAc\text{-}VP}$ projections in response to stimuli were not due to a movement artifact.

a, Strategy for recording eGFP fluorescence of D1$^{NAc\text{-}VM}$ or D1$^{NAc\text{-}VP}$ projection in response to stimuli in freely moving mice. *AAV$_9$-EF1α-DIO-eGFP* was injected into the NAc of *D1-Cre* mice, with optical fiber implanted over the VM or VP. **b-g**, Plot graph of group average eGFP signal aligned to the onset of sucrose licking (**b**), sucrose pellet consumption (**c**), sniffing of female stranger (**d**), retreat from the novel object in the central arena in the approaching-retreat test (**e**), air puff (**f**), or tail suspension (**g**). This control experiment was conducted to ascertain the validity of GCaMP6m fluorescence change in the projection in response to stimuli. Related to Figure 1.
