# Supplementary material for: A distinct D1-MSN subpopulation down-regulates dopamine to promote negative emotional state
Source: Cell Res. 2021 Nov 30;32(2):139–56. doi: 10.1038/s41422-021-00588-5 (PMC8807621; doi:10.1038/s41422-021-00588-5)
Supplement: Supplementary file 2 — Supplementary Figure 2 [file 41422_2021_588_MOESM2_ESM.pdf]

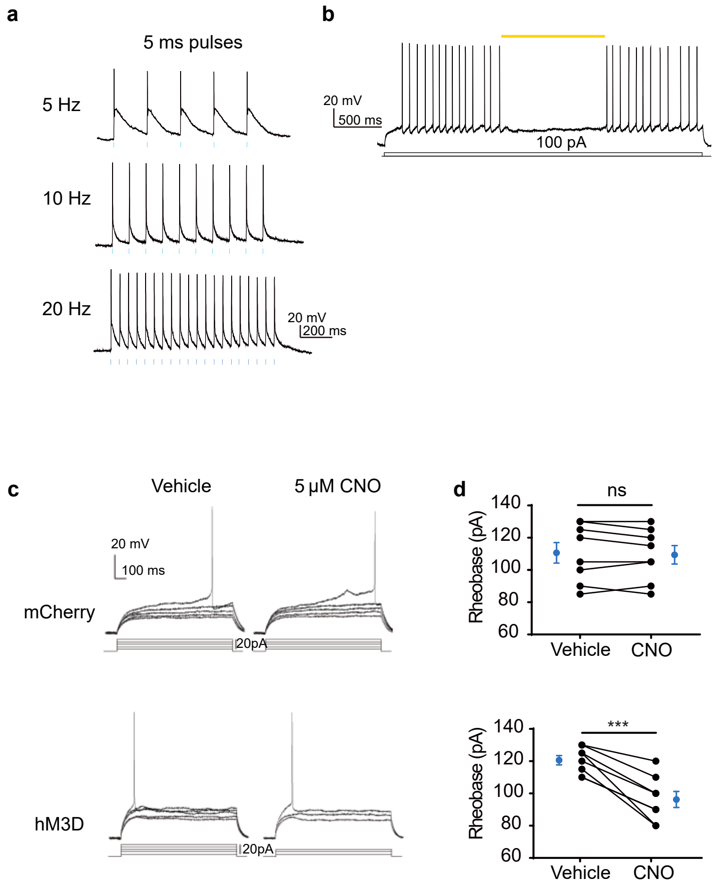
a
5 ms pulses
5 Hz
10 Hz
20 Hz
20 mV
200 ms
b
20 mV
500 ms
100 pA
c
Vehicle
5 µM CNO
20 mV
100 ms
mCherry
20pA
hM3D
20pA
d
ns
Rheobase (pA)
140
120
100
80
60
Vehicle
CNO
***
Rheobase (pA)
140
120
100
80
60
Vehicle
CNO

**Supplementary information, Fig. 2 *Ex vivo* Electrophysiological verification of optogenetic and chemogenetic stimulation.**

**a,** Action potentials generated by optical stimulation in accumbal D1-MSNs (5 Hz (top), 10 Hz (middle), 20 Hz (bottom), 473 nm, 2 mW, 5 ms pulse width, 1s duration). **b,** Representative voltage response evoked by 100-pA current injection showed that eNpHR3.0-expressing D1-MSNs were responsive to light inhibition (594 nm, 2 mW, 1s). **c,** Action potentials recorded from hM3D-expressing D1-MSN (n = 8) in response to progressively higher depolarizing currents before (baseline) and 15 min after CNO application. **d,** Rheobase decreased 15 min after CNO application. [mCherry: Paired *t*-test $t(7) = 0.798$, $p = 0.451$; hM3D: Paired *t*-test $t(7) = 6.363$, $p = 0.0004$] $^{***}p < 0.001$. Related to Figure 2 and Extended Data Figure 6.
