# Supplementary material for: A distinct D1-MSN subpopulation down-regulates dopamine to promote negative emotional state
Source: Cell Res. 2021 Nov 30;32(2):139–56. doi: 10.1038/s41422-021-00588-5 (PMC8807621; doi:10.1038/s41422-021-00588-5)
Supplement: Supplementary file 3 — Supplementary Figure 3 [file 41422_2021_588_MOESM3_ESM.pdf]

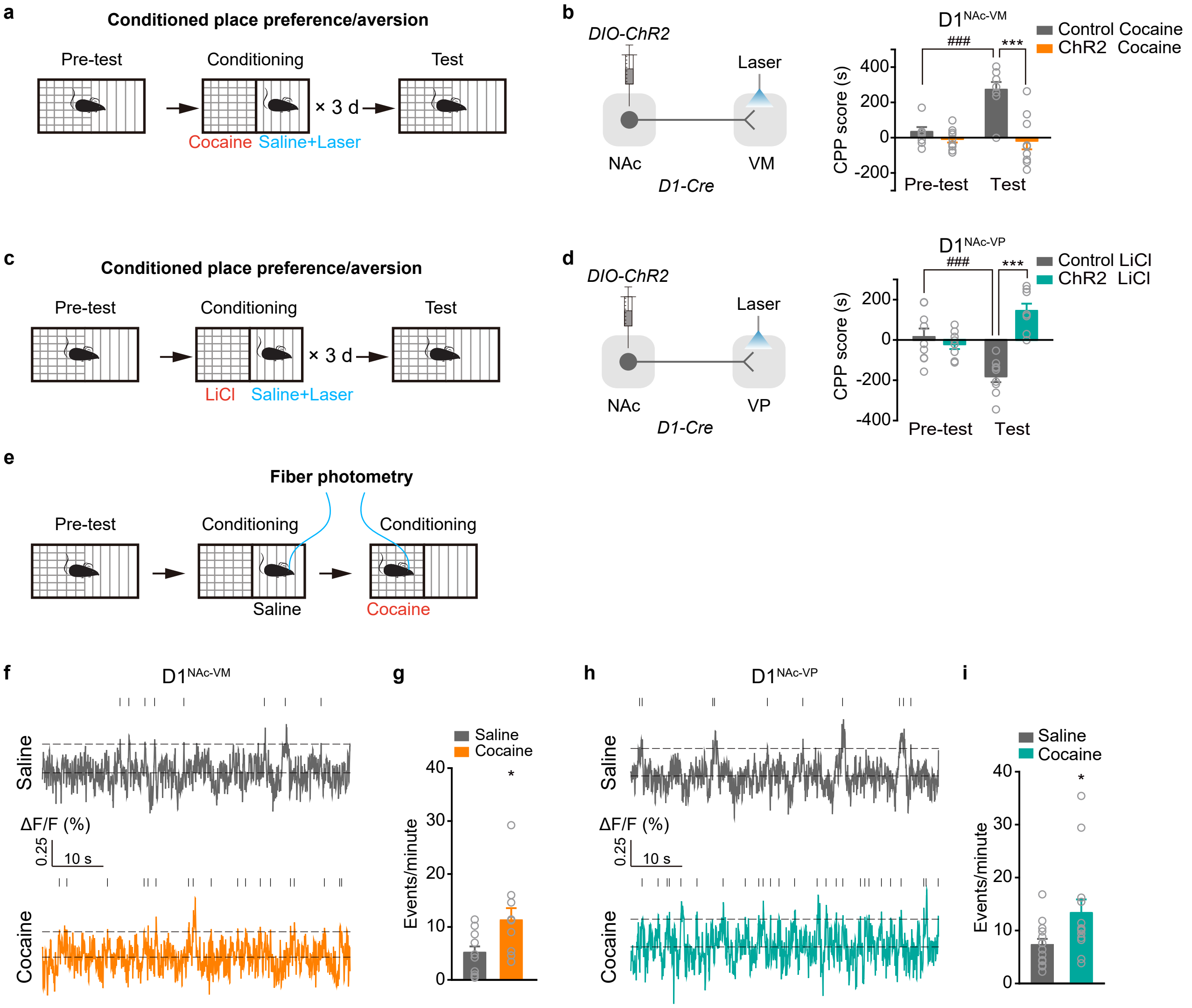
a
Conditioned place preference/aversion
Pre-test
Conditioning
Test
× 3 d
Cocaine
Saline+Laser
b
DIO-ChR2
Laser
NAc
VM
D1-Cre
D1NAc-VM
Control Cocaine
ChR2 Cocaine
###
***
CPP score (s)
400
200
0
-200
Pre-test
Test
c
Conditioned place preference/aversion
Pre-test
Conditioning
Test
× 3 d
LiCl
Saline+Laser
d
DIO-ChR2
Laser
NAc
VP
D1-Cre
D1NAc-VP
Control LiCl
ChR2 LiCl
###
***
CPP score (s)
200
0
-200
-400
Pre-test
Test
e
Fiber photometry
Pre-test
Conditioning
Conditioning
Saline
Cocaine
f
D1NAc-VM
Saline
ΔF/F (%)
0.25
10 s
Cocaine
g
Saline
Cocaine
Events/minute
40
30
20
10
0
*
h
D1NAc-VP
Saline
ΔF/F (%)
0.25
10 s
Cocaine
i
Saline
Cocaine
Events/minute
40
30
20
10
0
*

Supplementary information, Fig. 3 Cocaine conditioning increases frequency of Ca2+ transient events in both D1^{NAc-VM} and D1^{NAc-VP} projections.

a,c, Schematic of experimental design. *AAV9-EF1α-DIO-hChR2-mCherry* or *AAV9-EF1α-DIO-mCherry* was injected into the NAc of *D1-Cre* mice, and optical fibers were bilaterally implanted over the VM or VP. Laser stimulation was presented during saline conditioning. Cocaine and LiCl was conditioned in the other side. After three-day conditioning, the CPP/A test was performed. b,d, Bar graphs of CPP score. [Two-way RM ANOVA, VM: Control n = 8, ChR2 n = 9, $F_{treatment \times session}$ (1, 33) = 11.258, p = 0.004 ; VP: Control n = 8, ChR2 n = 9, $F_{treatment \times session}$ (1, 33) = 31.94, p < 0.001.] $^{***}p < 0.001$. e, Schematic of experimental design. *AAV9-EF1α-DIO-GCaMP6m* was injected into the NAc of *D1-Cre* mice, with optical fiber implanted over the VM or VP. f,h, Representative photometry signal traces of D1-MSN axonal terminals in the VM (f) and VP (h) during saline and cocaine conditioning. Tick marks indicate events above threshold. g,i, Frequency of Ca^{2+} transient events in D1^{NAc-VM} (g) and D1^{NAc-VP} (i) terminals was calculated during 5-10 min after saline or cocaine treatment [Two-tailed *Student's t*-test, n = 11, $t(20) = -2.336$, p = 0.0282; Mann-Whitney U test, n = 13, Z = 2.052, p = 0.0387.] $^{*}p < 0.05$. Related to Figure 3.
