# Supplementary material for: A distinct D1-MSN subpopulation down-regulates dopamine to promote negative emotional state
Source: Cell Res. 2021 Nov 30;32(2):139–56. doi: 10.1038/s41422-021-00588-5 (PMC8807621; doi:10.1038/s41422-021-00588-5)
Supplement: Supplementary file 4 — Supplementary Figure 4 [file 41422_2021_588_MOESM4_ESM.pdf]

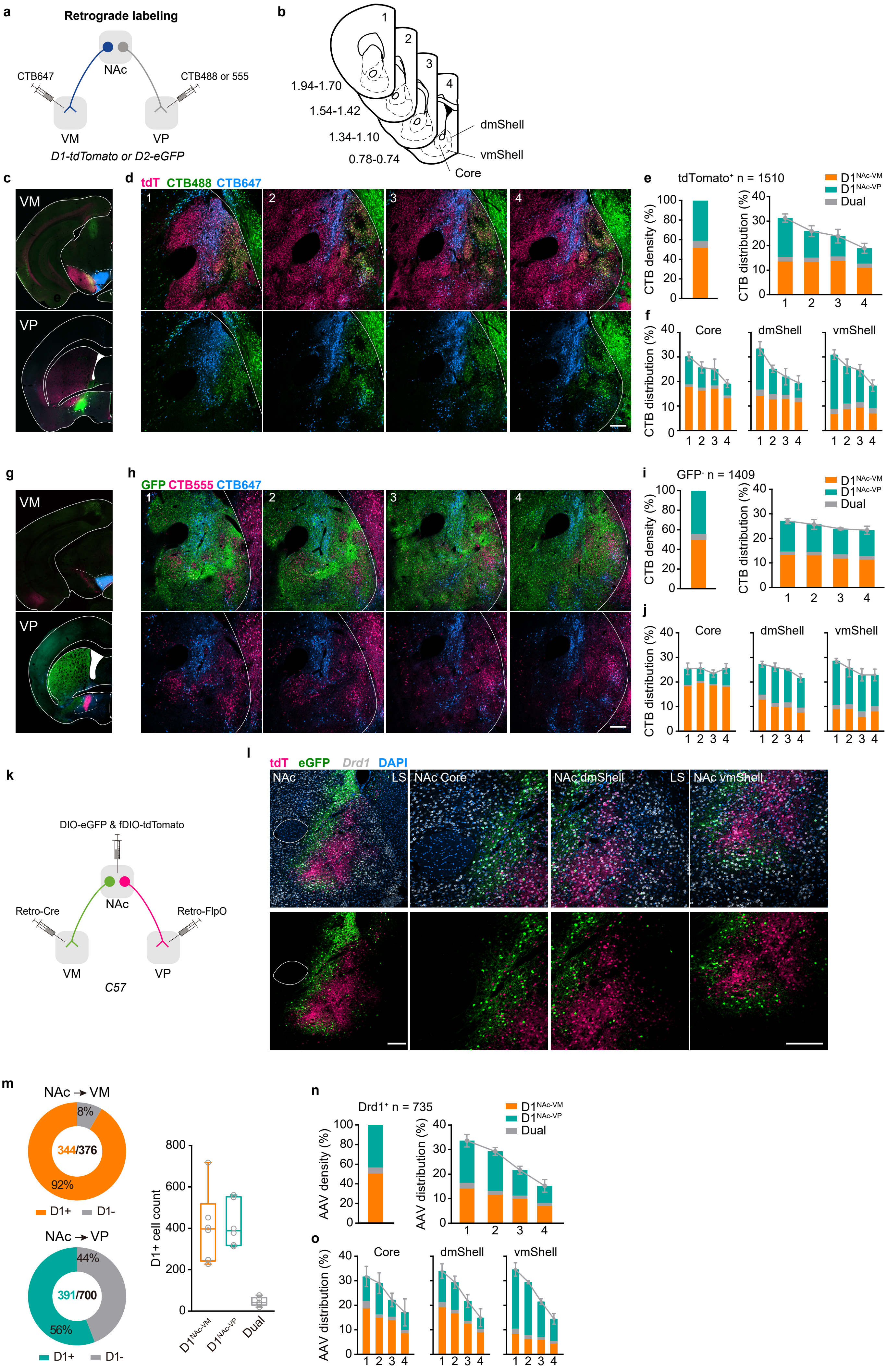

a
Retrograde labeling
NAc
CTB647
CTB488 or 555
VM
VP
D1-tdTomato or D2-eGFP
b
1.94-1.70
1.54-1.42
1.34-1.10
0.78-0.74
dmShell
vmShell
Core
c
d
tdT CTB488 CTB647
e
tdTomato+ n = 1510
D1 NAc-VM
D1 NAc-VP
Dual
CTB density (%)
CTB distribution (%)
f
Core
dmShell
vmShell
g
h
GFP CTB555 CTB647
i
GFP- n = 1409
j
k
DIO-eGFP & fDIO-tdTomato
Retro-Cre
Retro-FlpO
C57
l
tdT eGFP Drd1 DAPI
NAc
LS
NAc Core
NAc dmShell
NAc vmShell
m
NAc → VM
8%
344/376
92%
D1+
D1-
NAc → VP
44%
391/700
56%
D1+ cell count
D1 NAc-VM
D1 NAc-VP
Dual
n
Drd1+ n = 735
AAV density (%)
AAV distribution (%)
o

Supplementary information, Fig. 4 Retrograde labeling of D1$^{NAc\text{-}VM}$ and D1$^{NAc\text{-}VP}$ neurons in the NAc by CTB.

a, Schematic of CTB labeling in *D1-tdTomato* and *D2-eGFP* mice. **b,** Schemtic of sites imaged for quantification across the rostro-caudal gradient of NAc. c,g, Confocal images for injections of CTB647 into the VM and CTB488 into the VP of *D1-tdTomato* mice, and injections of CTB647 into the VM and CTB555 into the VP of *D2-eGFP* mice. d,h, Confocal images for CTB labeled neurons in the NAc of *D1-tdTomato* (d) and *D2-eGFP* mice (h). Scale bar: 100 μm. e,i, Bar graph shows average proportion of CTB647 and CTB488/555 neurons relative to total tdTomato$^+$ or eGFP$^-$ retrograde labeling neurons in the NAc [tdTomato$^+$: VM: 58.7% ± 4.1%, VP: 48.0% ± 4.4%, Dual: 6.7% ± 0.4%, n = 6 mice; eGFP$^-$: VM: 55.7% ± 2.2%, VP: 50.2% ± 2.8%, Dual: 5.9% ± 0.8%, n = 5 mice; h, CTB647: 789 ± 77 cells, CTB555: 700 ± 45 cells of 4 slices per mouse]. f,j, Distribution of CTB-labeled tdTomato$^+$ or eGFP$^-$ neurons in the whole NAc, NAc core, dmShell, and vmShell correspondingly across NAc rostro-caudal gradient. k, Schematic of viral strategy for dual-projection labeling of VP projecting and VM projecting NAc neurons in the same animal. l, Confocal images for *drd1* expression, tdTomato and eGFP labeled neurons in the NAc. Scale bar: 100 μm. m, Left: quantification of *drd1*$^+$ tdTomato$^+$ and *drd1*$^+$ eGFP$^+$ cell counts in the NAc. Right: summary of the number of tdTomato$^+$ D1$^-$MSNs, eGFP$^+$ D1$^-$MSNs and double positive D1-MSN in the NAc [VM = 406 ± 72 cells, VP = 420 ± 45 cells, Dual = 45 ± 8 cells of 4 slices per mouse]. $^*p < 0.05$. n, Bar graph shows proportion of *drd1*$^+$ tdTomato$^+$ and *drd1*$^+$ eGFP$^+$ neurons relative to total retrograde labeling D1-MSNs in the NAc [VM: 49.1% ± 1.8%, VP: 56.8% ± 2.2%, Dual: 5.8% ± 1.1%, n = 5 mice]. o, Distribution of retrograde labeling D1-MSNs across

NAc rostro-caudal gradient. Numbers represent number of labeled cells per mouse. Related to Figure 4.
