# Supplementary material for: A distinct D1-MSN subpopulation down-regulates dopamine to promote negative emotional state
Source: Cell Res. 2021 Nov 30;32(2):139–56. doi: 10.1038/s41422-021-00588-5 (PMC8807621; doi:10.1038/s41422-021-00588-5)
Supplement: Supplementary file 5 — Supplementary Figure 5 [file 41422_2021_588_MOESM5_ESM.pdf]

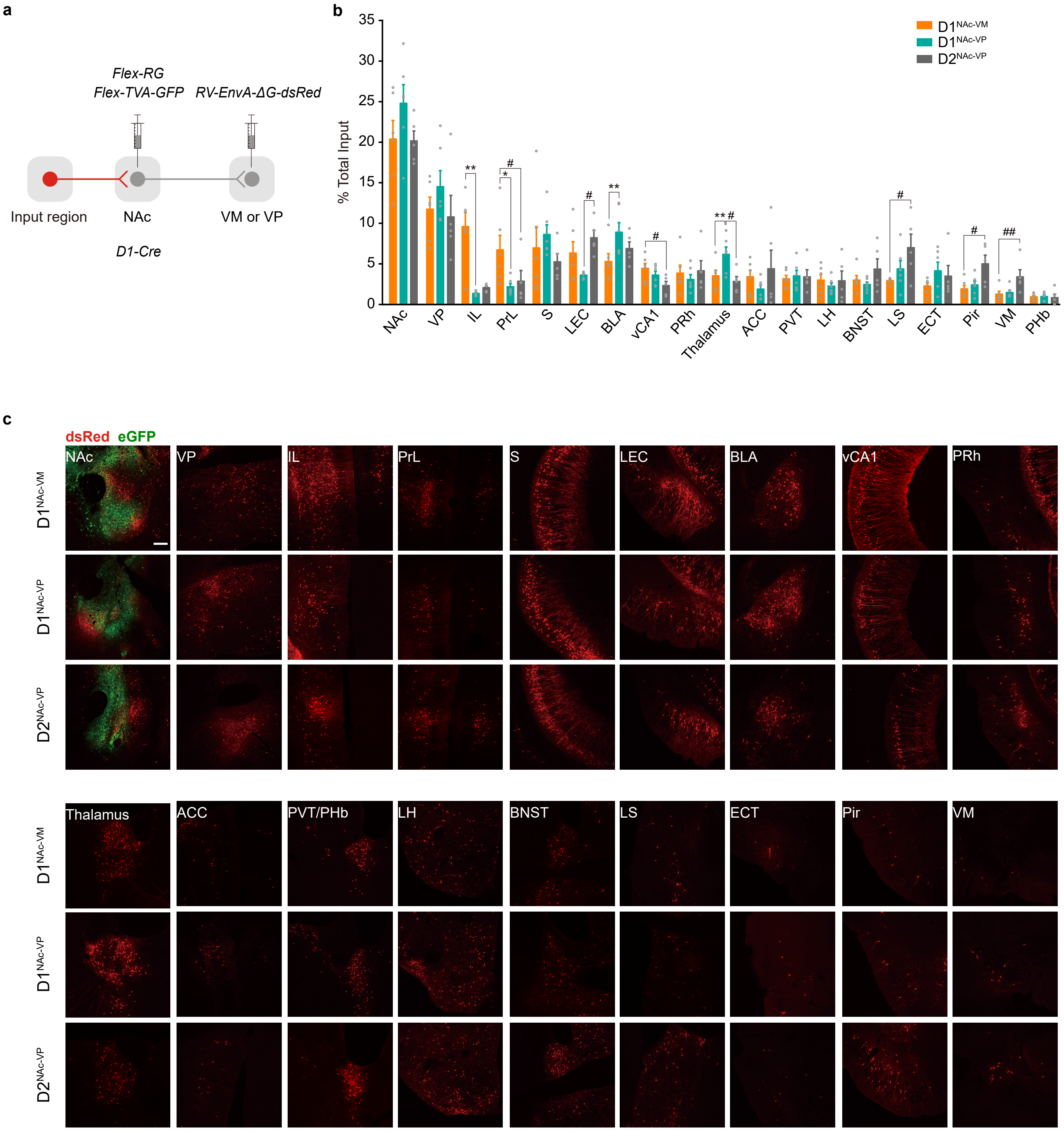

a
Flex-RG
Flex-TVA-GFP
RV-EnvA-ΔG-dsRed
Input region
NAc
VM or VP
D1-Cre
b
% Total Input
D1NAc-VM
D1NAc-VP
D2NAc-VP
NAc
VP
IL
PrL
S
LEC
BLA
vCA1
PRh
Thalamus
ACC
PVT
LH
BNST
LS
ECT
Pir
VM
PHb
c
dsRed
eGFP
PVT/PHb

Supplementary information, Fig. 5 Tracing inputs to D1$^{NAc\text{-}VM}$ and D1$^{NAc\text{-}VP}$ neurons defined by their output site.

a, Schematic of rabies virus-based monosynaptic tracing. *AAV9-EF1α-DIO-mCherry-F2A-TVA* and *AAV9-EF1α-DIO-RVG* were injected unilaterally in the NAc of *D1-* or *D2-Cre* mice. Two weeks later, *RV-ENVA-deltaG-dsRed* (RV*d*G) was injected into the VM or VP. b, Brain-wide quantification of inputs to D1$^{NAc\text{-}VM}$ and D1$^{NAc\text{-}VP}$ neurons [D1$^{NAc\text{-}VM}$ n = 6, D1$^{NAc\text{-}VP}$ n = 6, Two-tailed *Student's t*-test, PrL: $t(10) = 2.457$, $p = 0.034$; Thalamus: $t(10) = -2.293$, $p = 0.044$; Mann-Whitney U test, IL: Z = -2.882, $p = 0.0022$;BLA: Z = 2.242, $p = 0.026$.] $^{*}p < 0.05$, $^{**}p < 0.01$ vs D1$^{NAc\text{-}VP}$, $^{\#}p < 0.05$, $^{\#\#}p < 0.01$ vs D2$^{NAc\text{-}VP}$. Data are presented as a percentage of total input counted in each individual brain. Abbreviations of brain region shown in legend of Extended Date Fig. 4c, Representative images of different input brain regions. The dsRed^{+} cells represent individual neurons sending monosynaptic input to D1$^{NAc\text{-}VM}$ or D1$^{NAc\text{-}VP}$ neurons in the NAc. Scale bar: 100 µm. NAc, nucleus accumbens; VP, ventral pallidum; S, subiculum; IL, infralimbic cortex; PrL, prelimbic cortex; BLA, basolateral amygdala; LEC, lateral entorhinal cortex; vCA1, ventral hippocampal; PRh, perirhinal cortex; LS, lateral sepal nucleus; ECT, ectorhinal cortex; ACC, anterior cingulate cortex; BNST, bed nucleus of the stria terminalis; LH, lateral hypothalamus; Pir, posterior insular cortex; VM, ventral mesencephalon; PVT, paraventricular nucleus of the thalamus; PHb, perihabenular nucleus. Related to Figure 4.
