# Supplementary material for: A distinct D1-MSN subpopulation down-regulates dopamine to promote negative emotional state
Source: Cell Res. 2021 Nov 30;32(2):139–56. doi: 10.1038/s41422-021-00588-5 (PMC8807621; doi:10.1038/s41422-021-00588-5)
Supplement: Supplementary file 6 — Supplementary Figure 6 [file 41422_2021_588_MOESM6_ESM.pdf]

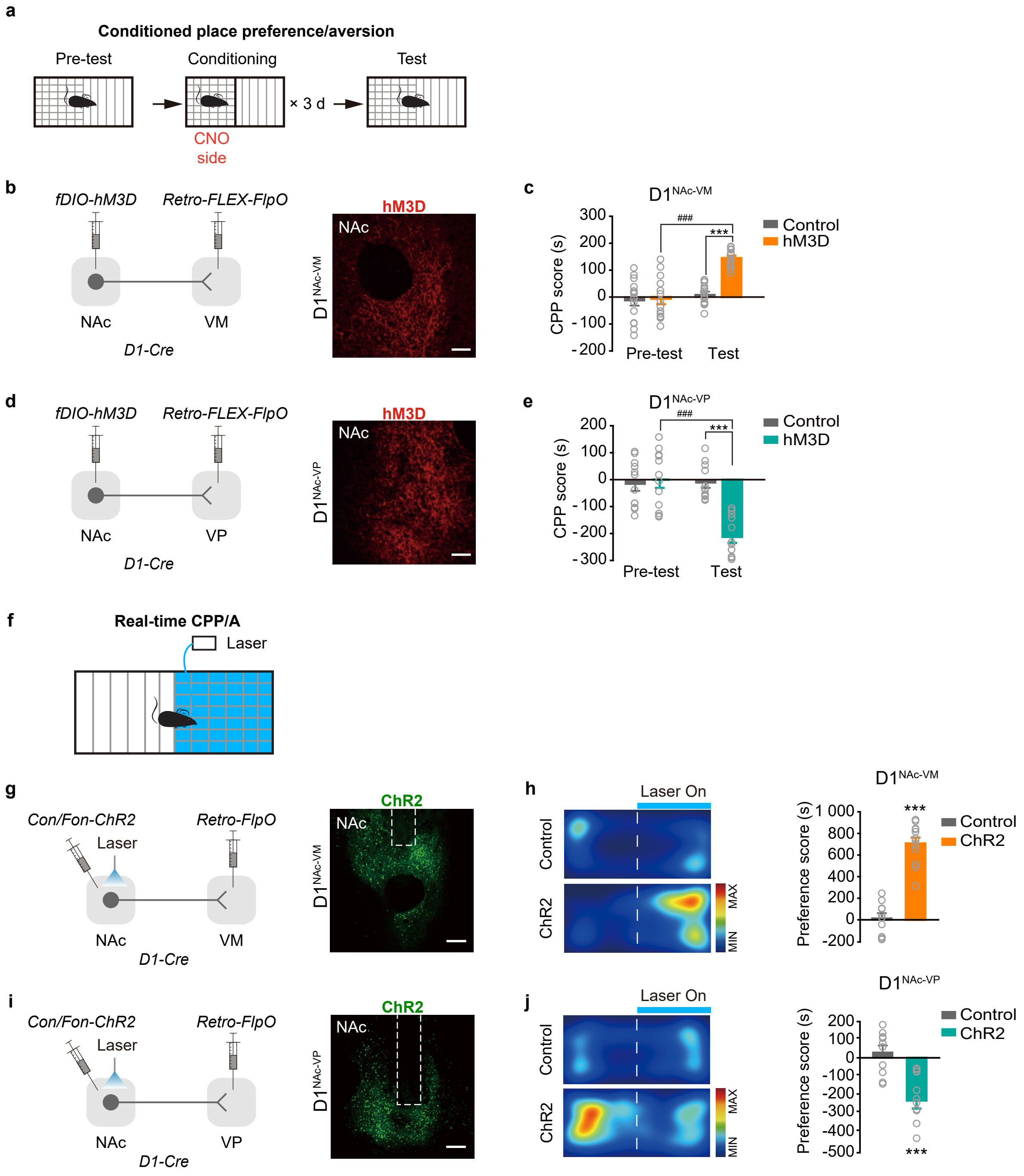
a
Conditioned place preference/aversion
Pre-test
Conditioning
Test
× 3 d
CNO side
b
fDIO-hM3D
Retro-FLEX-FlpO
NAc
VM
D1-Cre
hM3D
NAc
D1 NAc-VM
c
D1 NAc-VM
CPP score (s)
300
200
100
0
-100
-200
###
***
Pre-test
Test
Control
hM3D
d
fDIO-hM3D
Retro-FLEX-FlpO
NAc
VP
D1-Cre
hM3D
NAc
D1 NAc-VP
e
D1 NAc-VP
CPP score (s)
200
100
0
-100
-200
-300
###
***
Pre-test
Test
Control
hM3D
f
Real-time CPP/A
Laser
g
Con/Fon-ChR2
Laser
Retro-FlpO
NAc
VM
D1-Cre
ChR2
NAc
D1 NAc-VM
h
Laser On
Control
ChR2
MAX
MIN
D1 NAc-VM
Preference score (s)
1 000
800
600
400
200
0
-200
***
Control
ChR2
i
Con/Fon-ChR2
Laser
Retro-FlpO
NAc
VP
D1-Cre
ChR2
NAc
D1 NAc-VP
j
Laser On
Control
ChR2
MAX
MIN
D1 NAc-VP
Preference score (s)
200
100
0
-100
-200
-300
-400
-500
***
Control
ChR2

Supplementary information, Fig. 6 Activation of D1$^{NAc\text{-}VM}$ neurons produce CPP and activation of D1$^{NAc\text{-}VP}$ neurons produce CPA.

a, Schematic of CPP/A task. After three-day CNO paired conditioning, the CPP/A test was performed. **b,d,** Viral infection and representative images of hM3D-mCherry^{+} D1$^{NAc\text{-}VM}$ (**b**) or D1$^{NAc\text{-}VP}$ (**d**) neurons in the NAc. *AAV9-hEF1a-fDIO-hM3D-mCherry* was injected into the NAc and *AAV2/retro-CAG-FLEX-FlpO* was injected into the VM or VP of *D1-Cre* mice. Scale bar: 100 μm. **c,e,** Bar graphs of CPP score. [Two-way RM ANOVA. VM: Saline n = 15, CNO n = 14, $F_{\text{treatment×session}}$ (1,57) = 21.326, $p < 0.001$; VP: Saline n = 12, CNO n = 12, $F_{\text{treatment×session}}$ (1,47) = 67.686, $p < 0.001$]. $^{***}p < 0.001$ vs Control, $^{\#\#\#}p < 0.001$ vs Pre-test. **f,** Schematic of RTPP/A task. **g,i,** Viral infection and representative images of the optical fiber tip and ChR2-EYFP expression in the NAc. *AAV9-hSyn-Con/Fon-ChR2-EYFP* or *AAV9-hSyn-Con/Fon-EYFP* was injected into the NAc and *AAV2/retro-hSyn-FlpO* was injected into the VM or VP of *D1-Cre* mice. An optical fiber was implanted over the NAc and the RTPP/A test was performed. **h,j,** Representative locomotor heat maps of Test and bar graph of Preference score. [VM: Control n = 10, ChR2 n = 12, Two-tail *Student's t*-test, $t(20) = -9.406$, $p < 0.001$; VP: Control n = 10, ChR2 n = 12, $t(20) = 4.436$, $p = 0.0002$] $^{***}p < 0.001$. Related to Figure 4.
