# Supplementary material for: A distinct D1-MSN subpopulation down-regulates dopamine to promote negative emotional state
Source: Cell Res. 2021 Nov 30;32(2):139–56. doi: 10.1038/s41422-021-00588-5 (PMC8807621; doi:10.1038/s41422-021-00588-5)
Supplement: Supplementary file 7 — Supplementary Figure 7 [file 41422_2021_588_MOESM7_ESM.pdf]

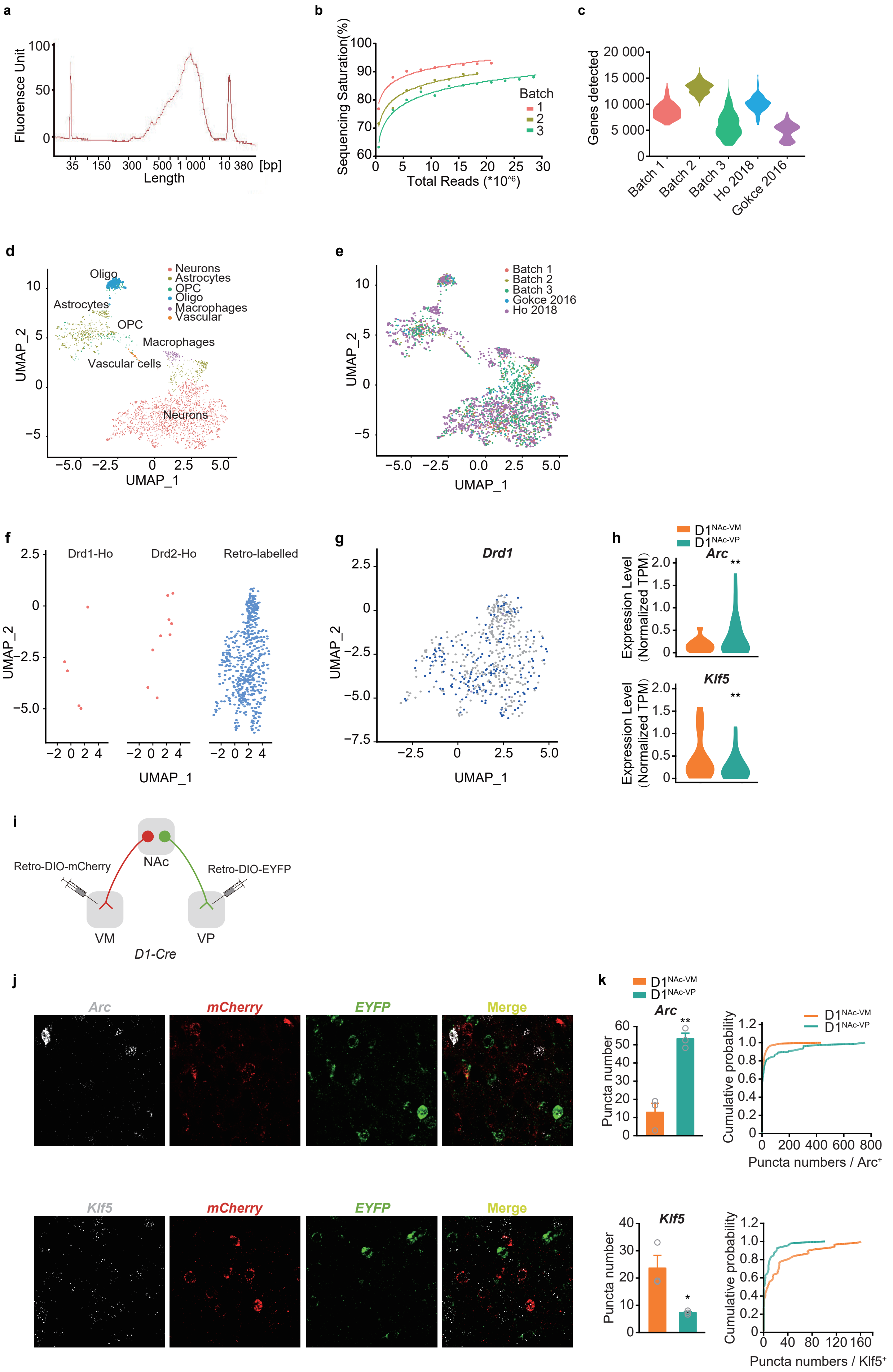
a
Fluorensce Unit
100
50
0
35
150
300
500
1 000
10 380
[bp]
Length
b
Sequencing Saturation(%)
100
90
80
70
60
0
5
10
15
20
25
30
Total Reads (*10^6)
Batch
1
2
3
c
Genes detected
20 000
15 000
10 000
5 000
0
Batch 1
Batch 2
Batch 3
Ho 2018
Gokce 2016
d
Neurons
Astrocytes
OPC
Oligo
Macrophages
Vascular
Oligo
Astrocytes
OPC
Macrophages
Vascular cells
Neurons
UMAP_2
UMAP_1
e
Batch 1
Batch 2
Batch 3
Gokce 2016
Ho 2018
UMAP_2
UMAP_1
f
Drd1-Ho
Drd2-Ho
Retro-labelled
UMAP_2
UMAP_1
g
Drd1
UMAP_2
UMAP_1
h
D1NAc-VM
D1NAc-VP
Arc
**
Expression Level
(Normalized TPM)
Klf5
**
i
Retro-DIO-mCherry
NAc
Retro-DIO-EYFP
VM
VP
D1-Cre
j
Arc
mCherry
EYFP
Merge
Klf5
mCherry
EYFP
Merge
k
D1NAc-VM
D1NAc-VP
Arc
**
Puncta number
Cumulative probability
Puncta numbers / Arc+
Klf5
*
Puncta numbers / Klf5+

Supplementary information, Fig. 7 Quality control of scRNA-Seq and correlation with previous RNA-Seq datasets of mouse striatum.

a, Representative example of PCR-amplified cDNA from one cell. **b,** Sequencing saturation plot from three batches of libraries prepared. The percentage of cumulative detected kmers from every 10k reads were plotted. **c,** Number of gene count distribution from each batch of samples, as well as striatal single cell data from Ho et al. (Ho 2018) and Gokce et al. (Gokce 2016). **d,** Distribution of data as colored by batch and origin. **e,** UMAP plot of combined single cell data from Ho *et al.* (Ho 2018) and Gokce *et al.* (Gokce 2016) and data we collected. **f,** Comparison of distribution of D1 and D2 cells from Ho *et al.* (Ho 2018) in neurons we collected. **g,** Distribution of D1-expressing cells in the neuronal population. **h**, Violin plot of *Arc* and *Klf5* expression in transcriptome analysis [Negative binomial generalized linear models, $D1^{NAc\text{-}VM}$ n = 120 cells, $D1^{NAc\text{-}VP}$ n = 182 cells, *Arc*: W = -2.743, $p = 0.007$; *Klf5*: W = 3.084, $p = 0.002$] **i,j**, Representative confocal images of smFISH of *EYFP* ($D1^{NAc\text{-}VP}$), *mCherry* ($D1^{NAc\text{-}VM}$) and *Arc* or *Klf5* (white) in the NAc of D1-Cre mice with the injection of *$AAV_{2/retro}$-EF1α-DIO-EYFP* into the VP and *$AAV_{2/retro}$-EF1α-DIO-mCherry* in the VM. Scale bar: 25 µm. **k**, Quantification bar graph and cumulative frequency distribution of *Arc* and *Klf5* expression in $D1^{NAc\text{-}VM}$ (*$EYFP^+$*) and $D1^{NAc\text{-}VP}$ (*$mCherry^+$*) neurons. [VM: n = 3, VP: n = 3, Two-tail *Student's t*-test, *Arc* :$t(4) = -6.831$, $p = 0.0024$; *Klf5* :$t(4) = 3.423$, $p = 0.0267$] $^{***}p < 0.001$ $^{*}p < 0.05$, $^{**}p < 0.01$. Related to Figure 4.
