# Supplementary material for: A distinct D1-MSN subpopulation down-regulates dopamine to promote negative emotional state
Source: Cell Res. 2021 Nov 30;32(2):139–56. doi: 10.1038/s41422-021-00588-5 (PMC8807621; doi:10.1038/s41422-021-00588-5)
Supplement: Supplementary file 8 — Supplementary Figure 8 [file 41422_2021_588_MOESM8_ESM.pdf]

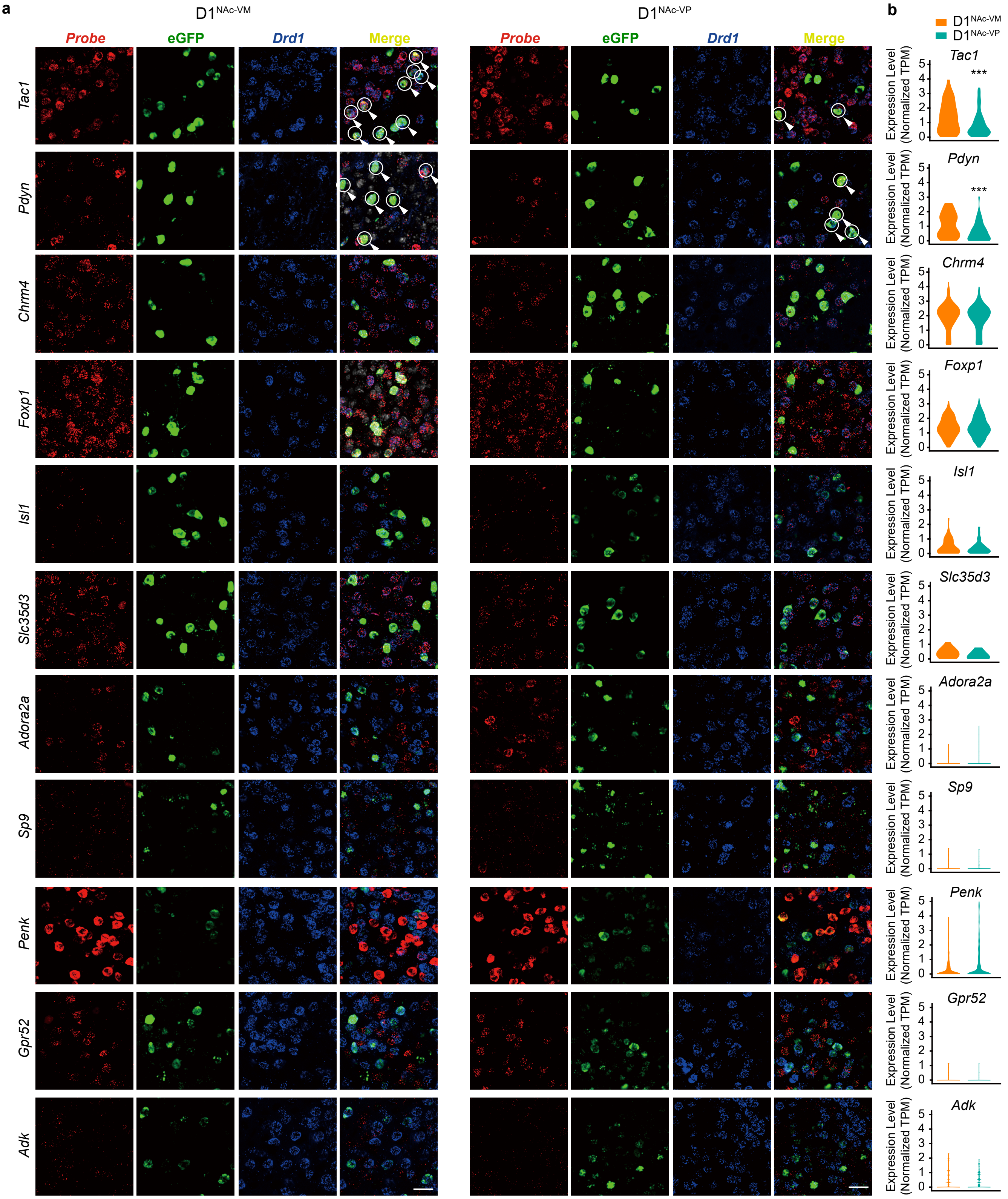

c

Expression level of MSN markers in D1^NAc-VM and D1^NAc-VP

Neuronal type	D1-MSN						D2-MSN				
	Tac1	*Pdyn*	*Chrm4*	*Foxp1*	*Isl1*	*Slc35d3*	*Adora2a*	*Sp9*	*Penk*	*Gpr52*	*Adk*
D1^NAc-VM	97.0±1.7	99.5±0.5	99.0±1.1	100.0±0.0	100.0±0.0	100.0±0.0	0.0±0.0	0.0±0.0	0.0±0.0	0.3±0.6	0.0±0.0
D1^NAc-VP	79.3±3.1**	77.0±5.1*	99.0±0.5	100.0±0.0	99.7±0.3	100.0±0.0	1.0±0.6	1.8±0.6	1.9±0.8	1.4±0.8	1.6±0.5

*$p < 0.05$, **$p < 0.01$.

Supplementary information, Fig. 8 Expression of D1- and D2-MSN markers in $D1^{NAc\text{-}VM}$ and $D1^{NAc\text{-}VP}$ neurons.

a, Representative confocal images of eGFP expression (Green), smFISH of the markers (red) and *Drd1* (Blue) in the NAc of C57 mice with the injection of *$AAV_{2/retro}$-hSyn-eGFP* into the VP or VM. Scale bar: 25 μm. **b,** Violin plot of D1-MSN and D2-MSN marker expression in transcriptome analysis [Negative binomial generalized linear models, $D1^{NAc\text{-}VM}$ n = 120 cells, $D1^{NAc\text{-}VP}$ n = 182 cells, see Statistical Table]. $^{***}p < 0.001$. **c,** Percentage of triple positive cells in the NAc out of $Drd1^+$ $eGFP^+$ cells. [Two-tailed *Student's t*-test, *Tac1*: $t(6) = -5.036$, $p = 0.00237$; *Pdyn*: $t(5) = -3.755$, $p = 0.013$]. $^{*}p < 0.05$, $^{**}p < 0.01$. Related to Figure 4.
