# Supplementary material for: A distinct D1-MSN subpopulation down-regulates dopamine to promote negative emotional state
Source: Cell Res. 2021 Nov 30;32(2):139–56. doi: 10.1038/s41422-021-00588-5 (PMC8807621; doi:10.1038/s41422-021-00588-5)
Supplement: Supplementary file 9 — Supplementary Figure 9 [file 41422_2021_588_MOESM9_ESM.pdf]

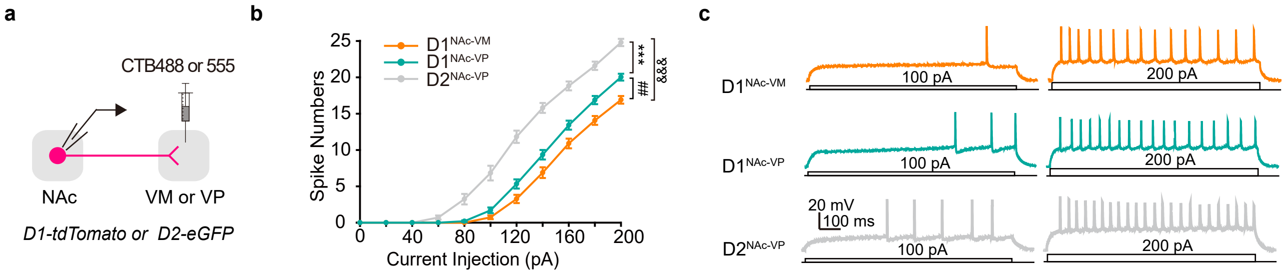

d

Intrisinc cellular excitability of $D1^{NAc-VM}$, $D1^{NAc-VP}$ and $D2^{NAc-VP}$

Measurement	$D1^{NAc-VM}$	$D1^{NAc-VP}$	$D2^{NAc-VP}$
Rheobase (pA)	128.5 ± 2.9	112.1 ± 2.5 ***	96.6 ± 2.5 $^{\&\&\&\#\#\#}$
AP threshold (mV)	-39.3 ± 0.3	-38.3 ± 0.4 *	-38.4 ± 0.4
Input Resistance (MΩ)	115.1 ± 3.9	114.8 ± 4.4	143.1 ± 6.8 $^{\&\#}$
Resting Potential (mV)	-76.8 ± 0.6	-75.2 ±0.7	-73.5 ± 0.8 *
Membrane Resistance (MΩ)	124.6 ± 3.0	127.6 ± 2.7	150.1± 4.3 $^{\&\#}$

$^{\#}p<0.05$, $^{\#\#\#}p<0.001$, $^{*}p<0.05$, $^{***}p<0.001$ vs $D1^{NAc-VM}$, $^{\&}p<0.05$, $^{\&\&\&}p<0.001$ vs $D1^{NAc-VP}$.

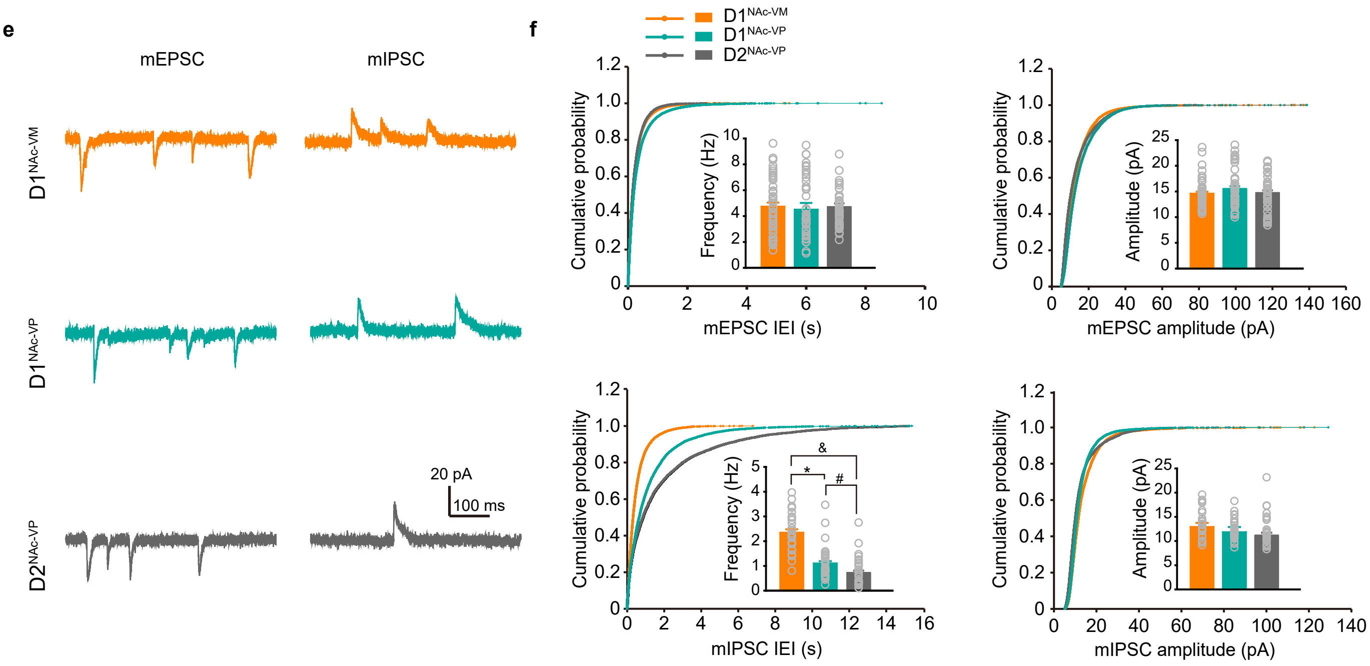

Supplementary information, Fig. 9 Electrophysiological properties of $D1^{NAc-VM}$ and $D1^{NAc-VP}$ neurons.

a, Schematic of electrophysiological recording of $D1^{NAc-VM}$, $D1^{NAc-VP}$, and $D2^{NAc-VP}$ neurons in *D1-tdTomato* or *D2-eGFP* mice injected with CTB488 or CTB555. **b,** Graph for numbers of spike firings at the indicated current steps. [RM ANOVA with Geisser-Greenhouse correction, $D1^{NAc-VM}$ n = 42 from 9 mice, $D1^{NAc-VP}$ n = 40 from 10 mice, $D2^{NAc-VP}$ n = 34 from 10 mice, $F_{type \times current}$ (20, 1130) = 25.353, $p < 0.001$.] $^{***}p < 0.001$, $^{\#\#}p < 0.01$, $^{\&\&\&}p < 0.001$ vs indicated group. **c,** Representative voltage traces recording. The voltage responses were evoked by 100-pA and 200-pA current injections. **d,** Diagram of intrinsic cellular excitability of $D1^{NAc-VM}$, $D1^{NAc-VP}$ and $D2^{NAc-VP}$ neurons [One-way ANOVA ,$D1^{NAc-VM}$ n = 42 from 9 mice, $D1^{NAc-VP}$ n = 40 from 10 mice, $D2^{NAc-VP}$ n = 34 from 10 mice, Rheobase: $F_{(2, 115)} = 34.965$, $p < 0.001$; Resting potential: $F_{(2, 115)} = 5.343$, $p = 0.006$; *Kruskal-Wallis* One-way ANOVA on Ranks, AP threshold: H = 10.793 with 2 degrees of freedom, $p = 0.005$; Membrane resistance: H = 21.417 with 2 degrees of freedom, $p < 0.001$;Input resistance: $D1^{NAc-VM}$ n = 41, $D1^{NAc-VP}$ n = 33, $D2^{NAc-VP}$ n = 34, H = 11.449 with 2 degrees of freedom, $p = 0.003$]. $^{*}p < 0.05$,$^{***}p < 0.001$, $^{\#}p < 0.05$, $^{\#\#\#}p < 0.01$, $^{\&}p < 0.05$, $^{\&\&\&}p < 0.001$ vs indicated group. **e,** Representative traces of mEPSCs (left) and mIPSCs (right). **f,** Graphs for cumulative probability and bar graphs for mean values of frequency (left) and amplitude (right) of mEPSCs (upper, $D1^{NAc-VM}$ n = 45 from 9 mice, $D1^{NAc-VP}$ n = 47 from 8 mice, $D2^{NAc-VP}$ n = 45 from 8 mice) and mIPSCs (lower, $D1^{NAc-VM}$ n = 36 from 8 mice, $D1^{NAc-VP}$ n = 39 from 9 mice, $D2^{NAc-VP}$ n = 36 from 7 mice). [*Kruskal-Wallis* One-way ANOVA on Ranks, H = 56.698 with 2 degrees of freedom, $p < 0.001$]. $^{*}p < 0.05$, $^{\#}p < 0.05$, $^{\&}p < 0.05$ vs indicated group. $^{*}p < 0.05$, $^{\#}p < 0.05$, $^{\&}p < 0.05$ vs indicated group. Related

to Figure 4.
