# Supplementary material for: A distinct D1-MSN subpopulation down-regulates dopamine to promote negative emotional state
Source: Cell Res. 2021 Nov 30;32(2):139–56. doi: 10.1038/s41422-021-00588-5 (PMC8807621; doi:10.1038/s41422-021-00588-5)
Supplement: Supplementary file 10 — Supplementary Figure 10 [file 41422_2021_588_MOESM10_ESM.pdf]

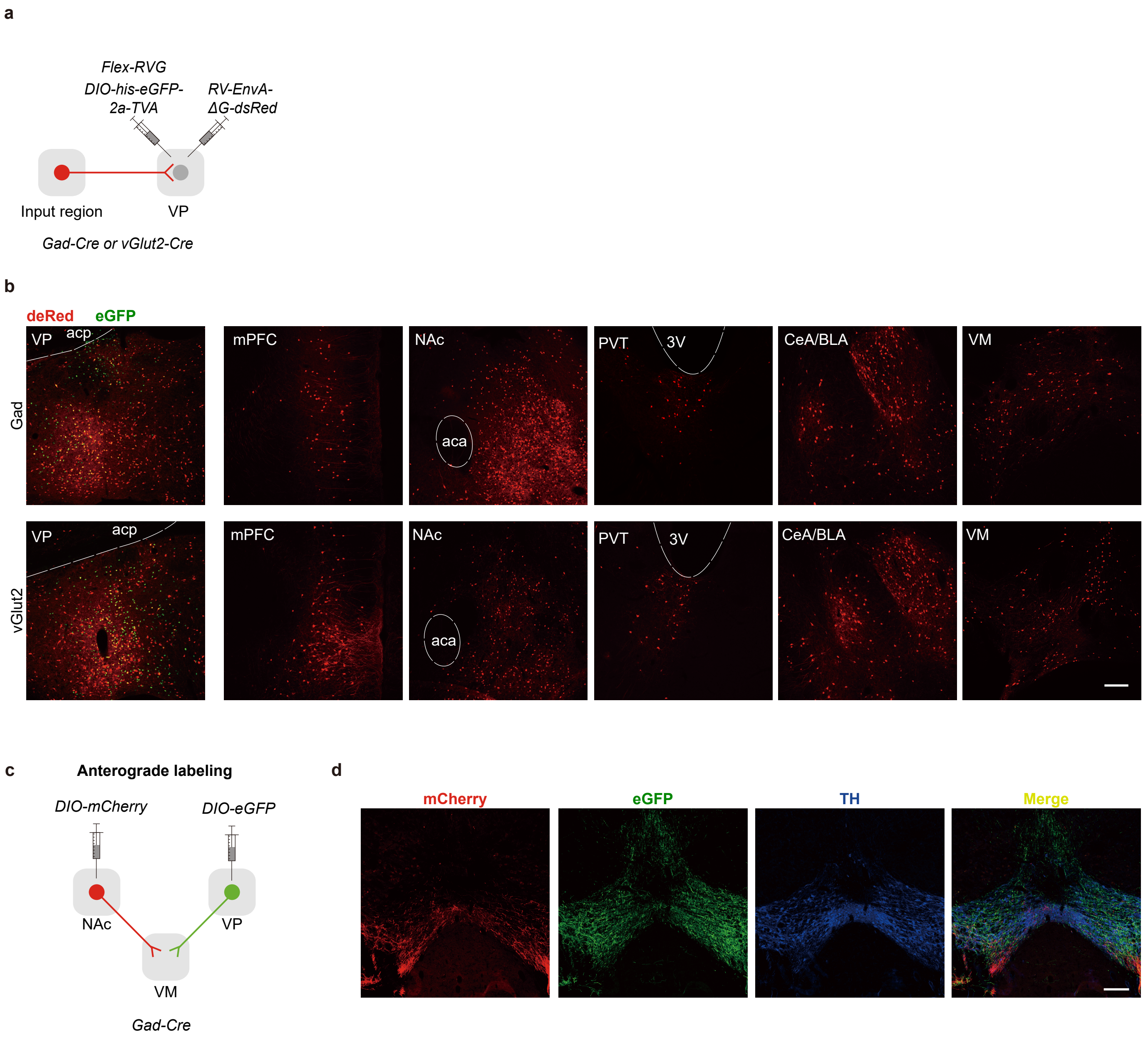
a
Flex-RVG
DIO-his-eGFP-
2a-TVA
RV-EnvA-
ΔG-dsRed
Input region
VP
Gad-Cre or vGlut2-Cre
b
deRed
eGFP
Gad
vGlut2
VP
acp
mPFC
NAc
aca
PVT
3V
CeA/BLA
VM
c
Anterograde labeling
DIO-mCherry
DIO-eGFP
NAc
VP
VM
Gad-Cre
d
mCherry
eGFP
TH
Merge

Supplementary information, Fig. 10 Rabies virus-based retrograde labeling of GABAergic or glutamatergic neurons in the VP.

a, Schematic of experimental design. *AAV9-EF1α-DIO-his-eGFP-2a-TVA* and *AAV9-EF1α-DIO-RVG* were unilaterally injected into the VP of *Gad-Cre* or *vGlut2-Cre* mice. Two weeks later, *RV-ENVA-deltaG-dsRed* (RV*d*G) was injected into the same VP site. Eight days were allowed for rabies to transduce and label synaptic connected input neurons. **b,** Representative confocal images of rabies labeling of given regions inputs to VP GABAergic or glutamatergic neurons from *Gad-Cre* or *vGlut2-Cre* mice, respectively. Scale bar: 100 μm. **c,** Schematic of anterograde labeling of $GABA^{NAc\text{-}VM}$ and $GABA^{VP\text{-}VM}$ projections. *AAV9-EF1α-DIO-mCherry* and *AAV9-EF1α-DIO-eGFP* were bilaterally injected into the NAc and VP of Gad-Cre mice. **d,** Representative images of $mCherry^+$ and $eGFP^+$ projectors in the VM with immunostaining of TH^+ neurons. Scale bar: 100 μm. Related to Figure 5.
